# Full-Length Transcriptome Sequencing and Comparative Transcriptomic Analyses Provide Comprehensive Insight Into Molecular Mechanisms of Cellulose and Lignin Biosynthesis in *Cunninghamia lanceolata*

**DOI:** 10.3389/fpls.2022.883720

**Published:** 2022-05-31

**Authors:** Xian-Ge Hu, Hebi Zhuang, Erpei Lin, Priyanka Borah, Mingqiu Du, Shiya Gao, Tongli Wang, Zaikang Tong, Huahong Huang

**Affiliations:** ^1^The State Key Laboratory of Subtropical Silviculture, Institute of Biotechnology, College of Forestry and Biotechnology, Zhejiang A&F University, Hangzhou, China; ^2^Department of Forest and Conservation Sciences, Faculty of Forestry, The University of British Columbia, Vancouver, BC, Canada

**Keywords:** *Cunninghamia lanceolata*, SMRT, full-length transcriptome, cellulose and lignin biosynthesis, transcription factor

## Abstract

*Cunninghamia lanceolata* is an essential timber species that provide 20%–30% raw materials for China’s timber industry. Although a few transcriptomes have been published in *C. lanceolata*, full-length mRNA transcripts and regulatory mechanisms behind the cellulose and lignin biosynthesis have not been thoroughly investigated. Here, PacBio Iso-seq and RNA-seq analyses were adapted to identify the full-length and differentially expressed transcripts along a developmental gradient from apex to base of *C. lanceolata* shoots. A total of 48,846 high-quality full-length transcripts were obtained, of which 88.0% are completed transcriptome based on benchmarking universal single-copy orthologs (BUSCO) assessment. Along stem developmental gradient, 18,714 differentially expressed genes (DEGs) were detected. Further, 28 and 125 DEGs were identified as enzyme-coding genes of cellulose and lignin biosynthesis, respectively. Moreover, 57 transcription factors (TFs), including *MYB* and *NAC*, were identified to be involved in the regulatory network of cellulose and lignin biosynthesis through weighted gene co-expression network analysis (WGCNA). These TFs are composed of a comparable regulatory network of secondary cell wall formation in angiosperms, revealing a similar mechanism may exist in gymnosperms. Further, through qRT-PCR, we also investigated eight specific TFs involved in compression wood formation. Our findings provide a comprehensive and valuable source for molecular genetics breeding of *C. lanceolata* and will be beneficial for molecular-assisted selection.

## Introduction

*Cunninghamia lanceolata* (Lamb.) Hook (Chinese fir) has been cultivated for over 1,000 years in southern China. As one of the essential coniferous species, *C. lanceolata* provides 20%–30% of China’s total commercial timber production (Huang et al., 2012). Further, the plant extracts of this species, which contains terpenoids, flavonoids, lignans, and essential oils, are used in traditional Chinese medicines to treat the traumatic injury, arthritis, and scabies diseases (Li et al., 2013, 2020; Zhao et al., 2015). Despite with long history of conventional breeding, the superior varieties of *C. lanceolata* with desirable wood quality are still scarce and cannot meet the market demands. At present, the molecular marker-assisted selection provides an effective strategy for breeding programmes of trees (Plomion et al., 1996; Lebedev et al., 2020; Yang et al., 2022). However, the shortage of genetic information severely limits the molecular-marker-assisted selection of *C. lanceolata*. Unfortunately, due to its large genome size (~11Gbp) and high heterozygosity (~2.0%; Huang et al., 2012; Lin et al., 2020b) the genome sequencing of *C. lanceolata* is still very challenging, costly and consume a lot of time.

In order to obtain more genetic information, high-throughput sequencing technologies have been increasingly applied in genome and transcriptome sequencing (Jiang et al., 2020; He et al., 2021). Several second-generation sequencing (SGS) reports on *C. lanceolata* have focused on SSR (simple sequence repeats) mining, gene identification, miRNA function and dormancy mechanisms (Huang et al., 2012; Cao et al., 2016; Jiang et al., 2020; Lin et al., 2020a). However, the short reads from SGS technology, which provided non-full-length sequences, probably resulted in incompletely assembled transcripts and loss of some vital information. Recently, the single-molecule real-time sequencing (SMRT) technology of PacBio RS system offers a new third-generation sequencing platform, which possesses advantages like long read lengths (length > 10 kb), high consensus accuracy and a low degree of bias. This platform has been successfully applied to sequence transcriptomes and identify functional genes of a specific biosynthetic pathway in gymnosperms, including *Ginkgo biloba* (Ye et al., 2019), *Pinus massoniana* (Mei et al., 2020) and *Pseudotaxus chienii* (Deng et al., 2020). Therefore, for those gymnosperms with a large and complicated genome, SMRT sequencing technology is an available and reliable strategy to generate more accurate and comprehensive genetic information.

Cellulose and lignin are the first and second most abundant biopolymers in wood, respectively (Engelhardt, 1995; Zhong et al., 2000). Their structure and content are tightly related to the wood quality and production. These biopolymers are the essential raw materials of modern industries and have been widely consumed in renewable biofuel production. Cellulose and lignin biosynthesis pathways are complex processes comprising many biological events strictly regulated by corresponding genes. The involvement of these genes has been investigated in model plants, including *Arabidopsis thaliana* (Zhou et al., 2014) and *Poplar* (Yoshida et al., 2013), and most of these genes can be divided into two types, namely enzyme-coding and regulatory genes. The enzyme-coding genes are directly involved in the biosynthesis process and regulatory factors through binding themselves to cis-acting elements in promoters of enzyme-coding genes effectively trigger the biosynthetic steps (Yoon et al., 2015). The hierarchical regulatory networks for forming the secondary cell wall (including cellulose and lignin biosynthesis pathway) in angiosperm already have been revealed based on comprehensive molecular and genetic studies (Vanholme et al., 2019; Zhong et al., 2019). These enzyme-coding and regulatory genes involved in cellulose and lignin biosynthesis are precious genetic resources for molecular breeding in timber trees. However, only a few of these genes have been identified and confirmed in gymnosperms, especially in *C. lanceolata*.

In the current study, the SMRT sequencing was applied to construct a full-length transcriptome of *C. lanceolata* for the first time. In parallel with SMRT sequencing, an SGS was also performed to investigate the global gene expressions in different lignified stem segments. Further, enzyme-coding genes in cellulose and lignin biosynthesis were specifically analyzed. In addition, the regulatory genes associated with the hierarchical regulatory networks of cellulose and lignin biosynthesis pathways were identified through weighted gene co-expression network analysis (WGCNA). Furthermore, a compression wood experiment was performed to investigate the identified regulatory genes during wood formation. Collectively, our study not only enriched our knowledge of genetic information in *C. lanceolata*, but also provided a rich gene resource for molecular breeding of this timber species.

## Materials and Methods

### Plant Materials and Sample Preparation

The plant materials were collected from 2-year-old *C. lanceolata* clone “2015–2,” which is one of the superior clones with faster growth rate and better wood quality and has been widely cultivated as commercial forest in Zhejiang province of China (Zhuang et al., 2021). As shown in Supplementary Figure S1, five 2-cm segments from apex to base of the current-year shoots were sequentially collected and named as the first segment (S1), second segment (S2), third segment (S3), fourth segment (S4), and fifth segment (S5), respectively. Additionally, bark (BK) and xylem (XY) samples were also harvested from the base (2–3 cm) of the stem. Samples were collected from five plants and pooled as a biological replicate, and three biological replicates were prepared in this study. These samples were used for cellulose and lignin contents determination, sequencing, and quantitative real-time polymerase chain reaction (qRT-PCR).

### Detection of Lignification Level and Cellulose/Lignin Contents Determination

The lignification level of different stem segments was detected using phloroglucinol–HCl stain (Liljegren, 2010). As indicated in Supplementary Figure S1, the collected segments were fixed in 5% agarose G-10 (BIOWEST, France) and then were cut into 50-μm sections using an automatic vibrating microtome (LEICA VT1200S, German). The sections were stained with 1% phloroglucinol–HCl and photographed by stereomicroscope (Zeiss Stemi 508, German). The cellulose and lignin contents were measured using chemistry method of Van Soest et al. (1991). Briefly, the stem segment was ground into fine powder, which was equilibrated at 37°C before measurement. To obtain the neutral detergent fiber (cellulose, hemicellulose, and lignin), about 0.2 g fine powder was placed in a crucible and heated to boil in 25-ml neutral detergent solution (3% SDS, 1.86% EDTA, 0.68% Na_2_B_4_O_7_, 0.456% Na_2_HPO_4_, 1.0% 2-ethoxyethanol, pH 6.9 ~ 7.1) for 60 min. The solution was filtered and washed with hot distilled water followed by washing with cold acetone. The crucible was dried in a hot air oven for 2 h at 105°C. Then, the dried crucible with the sample was added with 25-ml acid detergent fiber (ADF) solution (2% CTAB in 1.0 M H_2_SO_4_) and heated to boil for 60 min, and the solution was filtered and washed with hot distilled water followed by washing with cold acetone once again. The ADF (cellulose and lignin) calculation was performed as follows:


$$ADF\left(\%\right)=\frac{\left((\mathrm{Weight of crucible}+\mathrm{ADF})-\mathrm{Weight of crucible}\right)}{\left(\mathrm{Weight of sample}\right)}\times 100$$

The remaining residue of ADF was added with 25 ml 72% H_2_SO_4_ in the same crucible and mix it well. The resulting solution was incubated at 20°C for 3 h and then filtered and obtained residue washed twice with hot distilled water. The residue was then dried in a hot air oven at 105°C for 4 h followed by igniting at 550°C in muffle furnace for 10 min. Cellulose and lignin were calculated as per the following equations:


$$Cellulose\;\left(\%\right)=\frac{\left((\begin{array}{l} \mathrm{Weight of crucible}+\\ \mathrm{ADF} \end{array})-\mathrm{Weight of crucible}+\mathrm{Lignin}\right)}{\left(\mathrm{Weight of sample}\right)}\times 100$$

Lignin(%)=((Weight of crucible+Lignin)−Weight of crucible+Lignin+Ash)(Weight of sample)×100

### RNA Isolation

Total RNA was isolated through the RNA prep Pure Plant kit (TIANGEN Biotech, China) based on the manufacturer’s instructions. This RNA was further processed for purity and concentration, and then the nucleic acid absorption peak was observed using a NanoDrop 2000 spectrophotometer (Nano-Drop, United States). Based on Agilent 2,100 Bioanalyzer (Agilent, USA), the RNA integrity was investigated, and 1% agarose gel electrophoresis was used to survey the genomic DNA contamination. The qualified RNA samples were used for library building and sequencing.

### RNA-seq Library Construction and Sequencing

RNAs from the five stem segments, bark and xylem, were used for RNA-seq library construction and sequencing, and three biological replicates were performed for each sample. Briefly, total RNA from each sample was processed with DNase I, and mRNA was enriched with oligo (dT) magnetic beads and fractured into short fragments through fragmentation buffer. First-strand cDNA was generated using First Strand Master Mix and SuperScript II reverse transcription (Invitrogen). With DNA polymerase I and RNaseH, the second-strand cDNA was further synthesized and purified through AMPure XP beads (Beckman, United States). After end repairing, poly (A) tail was added, and adaptor ligation was also done. Subsequently, cDNA fragments were enriched by PCR amplification. The final library was evaluated using the Qubit 2.0, Agilent 2,100 bioanalyzer instrument, and qRT-PCR. The qualified libraries were sequenced on the Illumina HiSeq X Ten platform. After sequencing, raw reads were further filtered to generate clean reads by removing adaptors, low-quality reads and reads containing more than 10% unknown nucleotides. The Q30 and GC contents of clean reads were calculated based on quality control analysis.

### SMRT Sequencing Library Preparation and Sequencing

The total RNA from five different lignified samples (S1, S2, S3, S4, and S5) was mixed in equal amounts to construct libraries for full-length transcriptome sequencing. SMARTer^®^ PCR cDNA synthesis kit (Takara, China) was used to synthesize the first-strand cDNA and prepare full-length cDNA. Blue Pippin^™^ Size Selection System (Sage Science, Beverly, MA) was used for the size selection of different sizes of cDNA libraries (1–2, 2–3, and > 3 kb). According to the instructions, three SMRT libraries were constructed using SMRTbell template prep kit 1.0 (Pacific Biosciences) and were sequenced through seven SMRT cells using PacBio Sequel platform. Raw reads were processed to generate error-corrected reads of inserts (ROIs) through the isoform sequencing (Iso-Seq) pipeline. Full-length non-chimeric (FLNC) transcripts were then identified based on the poly (A) tail signal and the 5′ and 3′ cDNA primers in ROIs. Subsequently, the FLNC reads were clustered using iterative clustering algorithm for error correction (ICE) to produce the cluster consensus isoforms. The cluster consensus reads were polished with non-full-length reads to obtain high-quality isoforms. To remove the redundant sequences, the final isoform sequences were further screened by using software CD-HIT with a threshold of 0.99 identities (Li and Godzik, 2006). A benchmarking universal single-copy orthologs (BUSCO) assessment was performed to estimate the completeness of this transcriptome (Simão et al., 2015).

### Functional Annotation of Full-Length Transcripts and Identification of Differentially Expressed Genes

For the functional annotation of full-length transcripts, BLASTX alignment with E-value of 1E-5 was performed against public protein databases, including the National Center for Biotechnology Information (NCBI) non-redundant protein (NR) database, the Swiss-Prot database, the Kyoto Encyclopedia of Genes and Genomes (KEGG) database, and the Cluster of Orthologous Groups of proteins (COG) database. Gene Ontology (GO) annotation was performed by using Blast2GO software (Conesa et al., 2005), and GO function categories were implemented using WEGO software (Ye, 2006).

The clean RNA-seq data were mapped back to the full-length transcripts generated from SMRT sequencing to detect the expressions of the gene. The quantification of gene expression levels was calculated by software RSEM *via* transcripts per million (TPM) values (Li and Dewey, 2011), considering the effects of each sample’s gene length and sequencing depth. Then the differentially expressed genes (DEGs) between the stem segments were identified by using the DESeq R package (version 1.10.1) with screening criteria of fold change ≥ 2 and a false discovery rate (FDR) < 0.01 (Varet et al., 2016). The identified DEGs were annotated by GO and KEGG database and used for downstream analysis.

### Gene Co-expression Network Analysis and Visualization

Co-expression and module identification were implemented based on WGCNA in R software (Langfelder and Horvath, 2008). Modules were acquired through the automatic network established function (blockwise modules). The eigengene value was estimated for each cluster and utilized to investigate the association with cellulose and lignin. In addition, the connectivity of overall and intramodular kME (for modular membership) and *p* values were computed for the DEGs. Hub genes from the cellulose and lignin biosynthesis network were distinguished through Cytoscape plugin cytoHubba and ranked based on the maximal clique centrality values (Chin et al., 2014).

### Quantitative RT-PCR Validation and Expression Analysis

Total RNA isolated from the samples described above was used as a template and reverse-transcribed using PrimeScript RT Reagent Kit (Takara, China). Primers utilized in this research were projected through Primer 5 and listed in Supplementary Table S1. qRT-PCR was performed with TB Green Fast qPCR Mix (Takara, China) on a CFX96 Touch Thermal Cycler (Bio-Rad) according to the manufacturer’s instruction. Relative expression of each gene was calculated according to a 2^−ΔΔCt^ method with a housekeeping gene *Actin* used as an internal control (Zhang et al., 2019; Zhuang et al., 2021). The experiment was implemented with three biological and technical replicates.

### A Compression Wood Experiment to Investigate the Relationships Between Identified Regulatory Genes With Wood Formation

Reaction wood, which is called compression wood (CW) in gymnosperms, provides an excellent model experimental system for dissecting the molecular and genetic regulation of wood formation (Chao et al., 2019). In this study, the relationships between identified genes and wood formation were further checked through the compression wood experimental system. One-and-a-half-year-old plants of the clone 2015–2 were used as materials to induce compression woods by tilting the plants to ~45° from the vertical direction. The compression woods from five treated plants were harvested in the intervals of 30 days (CW 30 days) and 60 days (CW 60 days) for analysis. The normal woods from five untreated plants were also collected as a control (CK). Samples were collected from five plants and pooled as a biological replicate, and three biological replicates were prepared in this study. The samples were stored in fixative [4% (V/V) paraformaldehyde and 0.05% glutaraldehyde solute in 25 mM phosphate buffer] overnight, followed by washing/dehydration in ethanol and embedding in LR White acrylic resin (LR White, England). Embedded wood samples were sectioned in the transverse plane at 4-μm thickness with a glass knife (Leica RM2265, Germany), and sections were transferred to silane adhesive coated microscope slides. Stained with 1% safranin aqueous solution (for microscopy, SIGMA) and photographed (Leica DM4000 M, Germany); to detect lignin in the same section, lignin autofluorescence of the section was observed by a laser confocal microscope (ZEISS LSM880, Germany).

### Statistical Analysis

Statistical analysis was performed using Data Processing System Software (V16.05; Tang and Zhang, 2013) and differences were considered significant at *value of p* < 0.05 level. Statistical analyses for contents of cellulose and lignin were performed by using the least significant difference (LSD) test. Comparison among results of qRT-PCR was done by using Duncan test.

## RESULTS

### Quantification of Cellulose and Lignin in the Stem of *Cunninghamia lanceolata*

The lignification degree of different stem segments was first detected using phloroglucinol stain. The dyed area gradually enlarged and became deeper from S1 to S5 (Supplementary Figure S1), indicating an increase in lignification degree from stem apex to base. Further quantitative analysis of the S1 to S5 showed that the cellulose content increased from 15.64% to 29.52%, and the lignin content also increased from 8.32% to 15.91% (Table 1), which was consistent with the staining results. Meanwhile, the multiple comparison analysis showed that the cellulose and lignin contents had significant differences across stem apex to base (Table 1) except for cellulose in S3 and S4 and lignin in S1 and S2. These results indicated that the different stem segments had various lignification levels and were suitable for further transcriptome sequencing and identifying genes involved in cellulose and lignin biosynthesis.

**Table 1:** Relative abundances of cellulose and lignin contents.

Samples^1^	Cellulose (%) ± SD^2^	Lignin (%) ± SD^2^
S1	15.64 ± 0.87 d^3^	8.32 ± 0.85 d
S2	20.24 ± 0.50 c	9.72 ± 1.53 cd
S3	24.90 ± 2.36 b	11.73 ± 0.90 bc
S4	27.78 ± 2.43 ab	12.89 ± 0.98 b
S5	29.52 ± 1.71 a	15.91 ± 2.02 a

1 :S1, S2, S3, S4, and S5 represent the five 2-cm segments from stem apex to base of the current-year shoots.

2 :The standard deviation among the biological repeat.

3 :Values within the same column followed by different lower-case letters are significantly different according to LSD test (*p* < 0.05).

### Development of *Cunninghamia lanceolata* Stem Full-Length Transcripts Through SMRT Sequencing

To obtain a wide coverage of *C. lanceolata* transcriptome, a pooled high-quality RNA from five samples (S1, S2, S3, S4, and S5) was sequenced using the PacBio Sequel platform. A total of 1,050,363 polymerase reads (~20.00 Gb data) were obtained. After removal of the adaptor and low-quality reads, a total of 10,078,821 subreads were further generated. The average length of the reads in each SMRT cell was 3,051, 2,680, 2,204, 1,413, 2,205, 1,326, and 2,225 bp, respectively (Supplementary Table S2). The numbers of ROI with high accuracy (read quality > 0.80) from the three libraries (1–2, 2–3, 3–6 kb) were 245,545, 266,549, and 211,981, respectively (Table 2; Supplementary Table S3). The comprehensive analysis of ROI sequences length showed that most of the ROI was in line with library size (Supplementary Figure S2A). Statistically, the numbers of full-length non-chimeric reads obtained from the different libraries were 205,449, 232,441 and 178,888, respectively (Table 2). Finally, the number of full-length non-chimeric reads was 616,778, with an average size of 2,450 bp (Table 2). Based on further sequence clustering by ICE and removing redundant sequences by CD-HIT (Supplementary Figure S2B), a total of 197,662 isoforms were obtained, of which high-quality full-length transcripts were 48,846. Integrity assessment of the high-quality full-length transcriptomes using BUSCO showed a total of 1,614 BUSCO groups, in which 1,420 (88.0%) are complete BUSCOs (included 841 single-copy and 579 duplicated BUSCOs), the missing and fragmented BUSCOs were 135 (8.3%) and 59 (3.7%), respectively.

**Table 2:** Summary of transcripts for *Cunninghamia lanceolata*.

cDNA size	Reads of insert	Poly-A reads		Full-length reads			Full-length non-chimeric reads	
				Number	Percentage		Number	Average length (bp)
1–2 k	245,545	224,015		213,662	0.87		205,449	1,411
2–3 k	266,549	252,564		238,893	0.90		232,441	2,342
3–6 k	211,981	196,998		179,441	0.85		178,888	3,598
Total	724,075	677,036		682,131	0.87		616,778	2,450

The high-quality full-length transcripts of *C. lanceolata* were annotated based on the database of Nr, Swiss-Prot, COG, GO and KEGG (Table 3). A total of 43,376 transcripts (88.80%) were annotated for function, among which 41,150 and 37,596 transcripts were assigned to Nr and Swiss-Prot databases, respectively (Table 3). Similarity analysis showed that transcripts of *C. lanceolata* had high homology to sequences of three major species (*Amborella trichopoda*, 18.3%; *Nelumbo nucifera*, 9.8%; *Vitis vinifera*, 4.3%; Supplementary Figure S3A). Further, 26,009 full-length transcripts were assigned to the COG classification, and the largest group was the cluster for general function prediction only (2,789; Supplementary Figure S3B). And, 26,466 transcripts were assigned to the GO database, which was classified into three main categories, including molecular function (22,775), biological process (15,443), and cellular component (4,810; Supplementary Figure S3C). In addition, pathway-based analyses showed that 7,384 full-length transcripts mainly were mapped to 128 KEGG pathways (Table 3). The largest pathway was the ribosome pathway (Ko03010), containing 127 transcripts, followed by the RNA transport pathway (Ko03013, 100 transcripts) and protein processing in the endoplasmic reticulum pathway (Ko04141, 77 transcripts; Supplementary Figure S3D).

**Table 3:** Summary of functional annotation of the *Cunninghamia lanceolata* transcriptome.

Database	Annotated number	300 ≤ length < 1,000	length ≥ 1,000
COG	26,009	424	25,585
GO	26,466	425	26,041
KEGG	7,384	227	7,157
Nr	41,951	801	41,150
Swissprot	37,596	623	36,973

### Identification of DEGs Between Stem Segments

**Figure 1:**
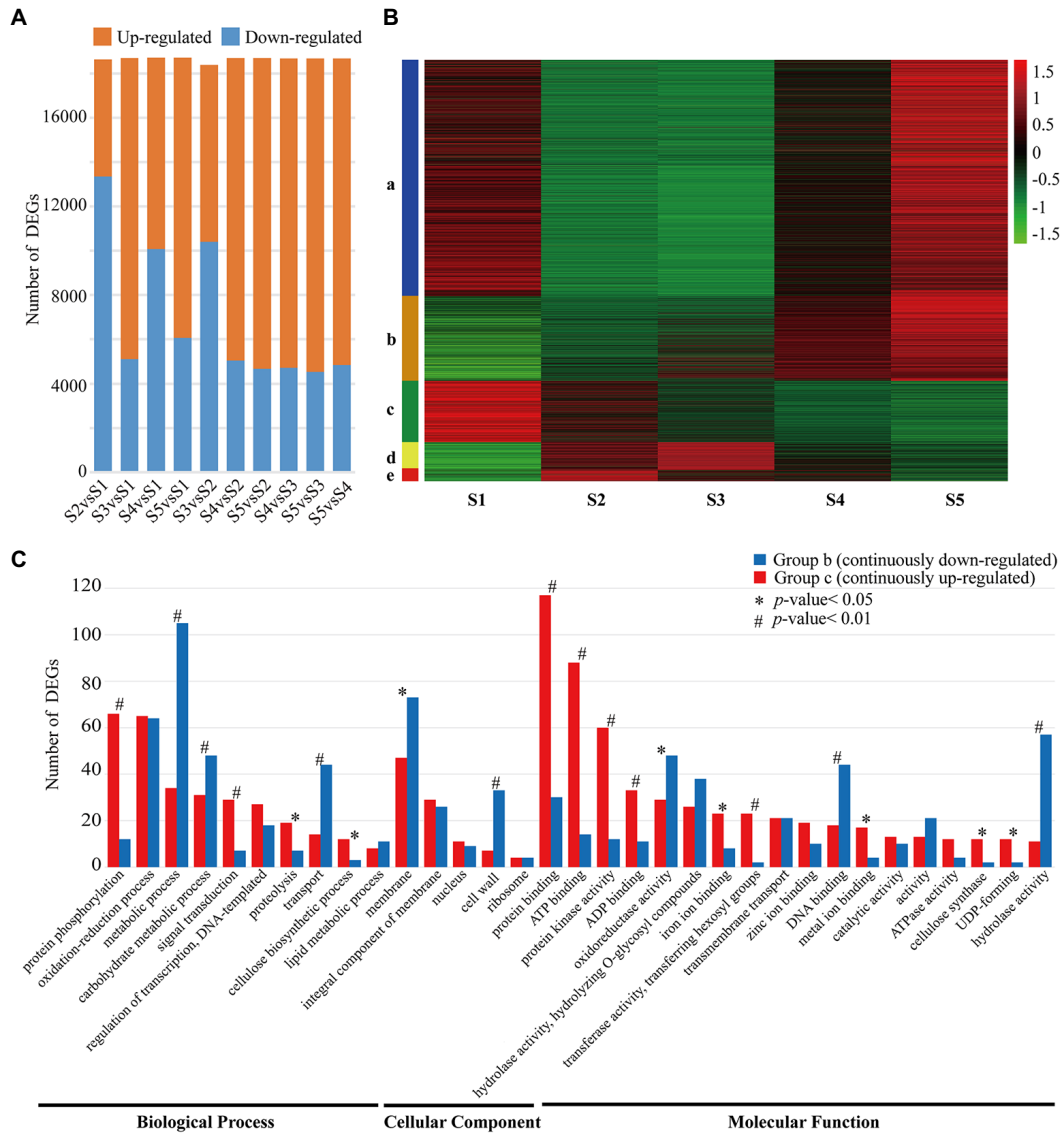
Profiling of DEGs between *Cunninghamia lanceolata* stem segments. **(A)** Number of DEGs between different samples. Upregulated and downregulated DEGs were marked with orange and blue, respectively. **(B)** Expression profiles of DEGs in five different samples. **(C)** GO classification of upregulated and downregulated DEGs. ^*^ and ^#^ indicate significant differences at 5% and 1%, respectively.

In order to identify the genes associated with cellulose and lignin biosynthesis, RNA samples from different stem segments were subjected to SGS. After filtration, 101 billion bases of clean data were obtained from SGS (Q30 > 87% and GC content > 31.9%). Then, by mapping these reads to the high-quality full-length transcripts (48,846) from SMRT sequencing, their expression levels in each sample were evaluated. Furthermore, 18,714 DEGs were identified between different stem segments (S1–S5) with false discovery rate (FDR) < 0.01 (Figure 1A).

As shown in Figure 1A when compared with the low lignified stem segments (S1), the relative number of upregulated and downregulated DEGs changed greatly with the increase in lignification, while the number of upregulated and downregulated DEGs remained stable between the high lignified stem segment (S3–S5). Meanwhile, the expressions of these DEGs could be grouped into five patterns including continuously upregulated (Figure 1B b) and continuously downregulated (Figure 1B c) from S1 to S5. And, there were 904 DEGs and 696 DEGs with continuously upregulated and downregulated expression patterns. Further GO functional analysis showed that the above-mentioned 1,600 DEGs could be categorized into 33 GO functional groups (Figure 1C), among which “protein binding,” “ATP binding,” and “protein phosphorylation” were the top three functional groups of continuously upregulated DEGs, whereas “metabolic process,” “cell wall,” and “hydrolase activity” were the top three functional groups of continuously downregulated DEGs (Figure 1C).

To verify DEGs’ expressions, 20 DEGs were randomly selected and analyzed using quantitative real-time PCR (qRT-PCR). Generally, the expression patterns of these DEGs obtained by analyzing RNA-Seq data were consistent with the results of qRT-PCR (Supplementary Figure S4). For example, the expressions of *CAD* (CL.67393.4), *CCR* (CL.65493.2), *COMT* (CL.67897.1), and *CCoAOMT* (CL.67870.5) were continuously upregulated from S1 to S5, which were further confirmed by qRT-PCR results (Supplementary Figure S4). And, the RNA-Seq data showed that *PAL* (CL.4241.14) and *SAD* (CL.58418.3) were continuously downregulated with the stem developmental gradient, which also were consistent with the results of qRT-PCR (Supplementary Figure S4). The consistency between qRT-PCR and RNA-seq data results indicates that the sequencing data were accurate and reliable.

### Enzyme-Coding Genes in the Cellulose Biosynthesis Pathway

As the primary cellulose biosynthetic pathways in *C. lanceolata* already had been characterized in our previous study (Huang et al., 2012), we further investigated the DEGs encoding specific enzymes of this pathway. Finally, a total of 28 DEGs across 7 gene families of the cellulose biosynthesis pathways were identified. The identified enzyme-coding genes included 19 *cellulose synthase subunits* (*CesA*) genes, three *Fructokinase* (*FRK*) genes, two *Sucrose synthase* (*SUS*) genes, one *Glucosyltransferase UDP-Glucose* (*GTF*) gene, one *Hexokinase* (*HK*) gene, one *Endo-1,4-beta-glucanase* (*KOR*) gene and one *Pyrophosphorylase* (*UPG*) gene (Table 4).

**Table 4:** Candidate differentially expressed genes involved in cellulose biosynthesis-related metabolism.

Gene	Enzyme	KO id (EC-No.)	No. all^a^	No. up^b^	No. down^c^	No. bark^d^	No. xylem^e^
CesA	Cellulose synthase	K10999 (EC:2.4.1.12)	19	5	1	0	5
SUS	Sucrose synthase	K00695 (EC:2.4.1.13)	2	0	0	0	0
GTF	Glucosyltransferase	K05841 (EC:2.4.1.173)	1	0	0	0	0
UGP	UDP-Glucose Pyrophosphorylase	K00963 (EC:2.7.7.9)	1	0	0	0	0
HK	Hexokinase	K00844 (EC:2.7.1.1)	1	0	0	0	0
FRK	Fructokinase	K00847 (EC:2.7.1.4)	3	1	0	0	0
KOR	Endo-1,4-beta-glucanase	K01179 (EC:3.2.1.4)	1	0	0	1	0

a Total gene number.

b Number of continuously upregulated genes from S1 to S5.

c Number of continuously downregulated genes from S1 to S5.

d Number of genes showed significant expression in bark compared to S1 to S5.

e Number of genes showed significant expression in xylems compared to S1 to S5.

**Figure 2:**
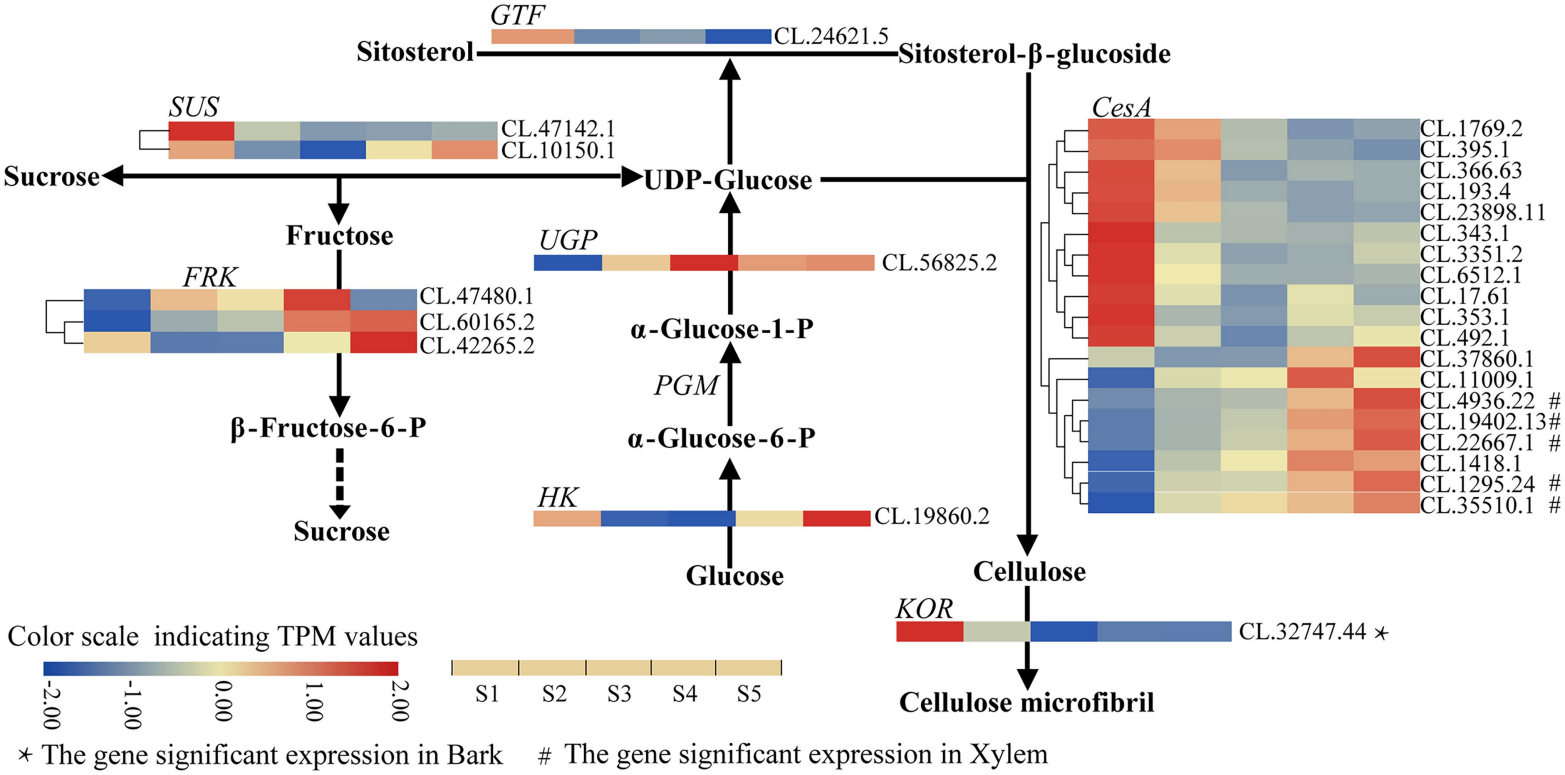
Heatmap depicting the expression profiles of DEGs involved in cellulose biosynthesis in *C. lanceolata*.

Further analysis indicated that the expression level of three *CesA* genes (CL.19402.13, CL.6512.1 and CL.23898.11) was higher (the average TPM > 100) than other enzyme-coding genes in stem developmental gradient of S1–S5 (Supplementary Table S4). With the enhancement of lignification (from S1 to S5), only one *CesA* gene (CL.395.1) was continuously downregulated. On the other hand, six genes [five *CesA* (CL.19402.13, CL.22667.1, CL.4936.22, CL.1295.24 and CL.35510.1) and one *FRK* (CL.60165.2)] were significantly continuously upregulated, in which five *CesA* genes also showed significant expression in xylem (Figure 2; Supplementary Table S4). It implies that these five *CesA* genes probably contribute to increasing cellulose content from S1 to S5. Additionally, the expression patterns of two *CesA* (CL.37860.1, CL.11009.1) and one *UGP* (CL.56825.2) changed irregularly from S1 to S5 but were dramatically upregulated in lignified stem segments (Figure 2; Supplementary Table S4). Interestingly, the identified upregulated *CesA* genes were clustered in family that genes have been proved to be related to secondary cell wall (SCW), and the downregulated *CesA* genes were clustered in gene family related to primary cell wall (PCW) formation (Supplementary Figure S5). In addition, *KOR* (CL.32747.44) was significantly expressed in bark (TPM value = 71.77) compared to the expression level in S1–S5 (Figure 2; Supplementary Table S4). Therefore, these DEGs with specific expression patterns were most likely associated with the cellulose biosynthesis in C*. lanceolata*.

### Enzyme-Coding Genes in the Lignin Biosynthesis Pathway

On the other hand, we also investigated the DEGs that encode enzymes involved in lignin biosynthesis pathways in *C. lanceolata* (Table 5). As a result, 125 DEGs across 13 gene families were identified as enzyme-coding genes of lignin biosynthesis, including four *Phenylalanine ammonia lyase* (*PAL*) genes, five *cinnamate 4-hydroxylase* (*C4H*) genes, 12 *4-coumarate CoA ligase* (*4CL*) genes, three *4-coumarate 3-hydroxylase* (*C3H*) genes, five *p-hydroxycinnamoyl CoA shikimate/quinate hydroxycinnamoyl transferase* (*HCT*) genes, two *Caffeoyl CoA O-methyltransferase* (*CCoAOMT*) genes, 10 *cinnamoyl CoA reductase* (*CCR*) genes, one *cinnamyl alcohol dehydrogenase* (*CAD*) genes, 22 *lactase* (*LAC*) genes, 52 *peroxidase* (*POD*) genes, six *caffeic acid O-methyltransferase* (*COMT*), one *Sinapyl alcohol dehydrogenase* (*SAD*) and two *S-adenosylmethionine synthetases* (*SAMS*; Table 5).

**Table 5:** Candidate differentially expressed genes involved in lignin biosynthesis-related metabolism.

Gene	Enzyme	KO id (EC-No.)	No. all^a^	No. up^b^	No. down^c^	No. bark^d^	No. xylem^e^
PAL	Phenylalanine ammonia-lyase	K10775 (EC:4.3.1.24)	4	0	0	0	2
C4H	Cinnamate 4-hydroxylase	K00487 (EC:1.14.14.91)	5	2	0	0	3
4CL	4-Coumarate CoA ligase	K01904 (EC:6.2.1.12)	12	1	0	1	0
C3H	4-Coumarate 3-hydroxylase	n.a.^f^ (EC:1.14.13)	3	0	0	0	2
HCT	p-Hydroxycinnamoyl CoA: shikimate/quinate hydroxycinnamoyl transferase	K13065 (EC:2.3.1.133)	5	0	0	0	1
CCoAOMT	Caffeoyl-CoA O-methyltransferase	K00588 (EC:2.1.1.104)	2	1	0	0	1
COMT	Caffeic acid O-mothyltransferase	K13066 (EC:2.1.1.68)	6	1	1	0	0
CCR	Cinnamoyl CoA reductase	K09753 (EC:1.2.1.44)	10	2	4	1	4
CAD	Cinnamoyl alcohol dehydrogenase	K00083 (EC:1.1.1.195)	1	1	0	0	0
SAD	Sinapyl alcohol dehydrogenase	K00083 (EC:1.1.1.195)	1	0	1	0	0
POD	Peroxidase	K00430 (EC:1.11.1.7)	52	2	11	0	8
SAMS	S-adenosylmethionine synthetase	K00789 (EC:2.5.1.6)	2	0	0	1	1
LAC	Lactase	K01229 (EC:3.2.1.108)	22	3	1	0	8

a Total gene number.

b Number of continuously upregulated genes from S1 to S5.

c Number of continuously downregulated genes from S1 to S5.

d Number of genes showed significant expression in bark compared to S1 to S5.

e Number of genes showed significant expression in xylems compared to S1 to S5.

f It is undefined in KEGG database.

**Figure 3:**
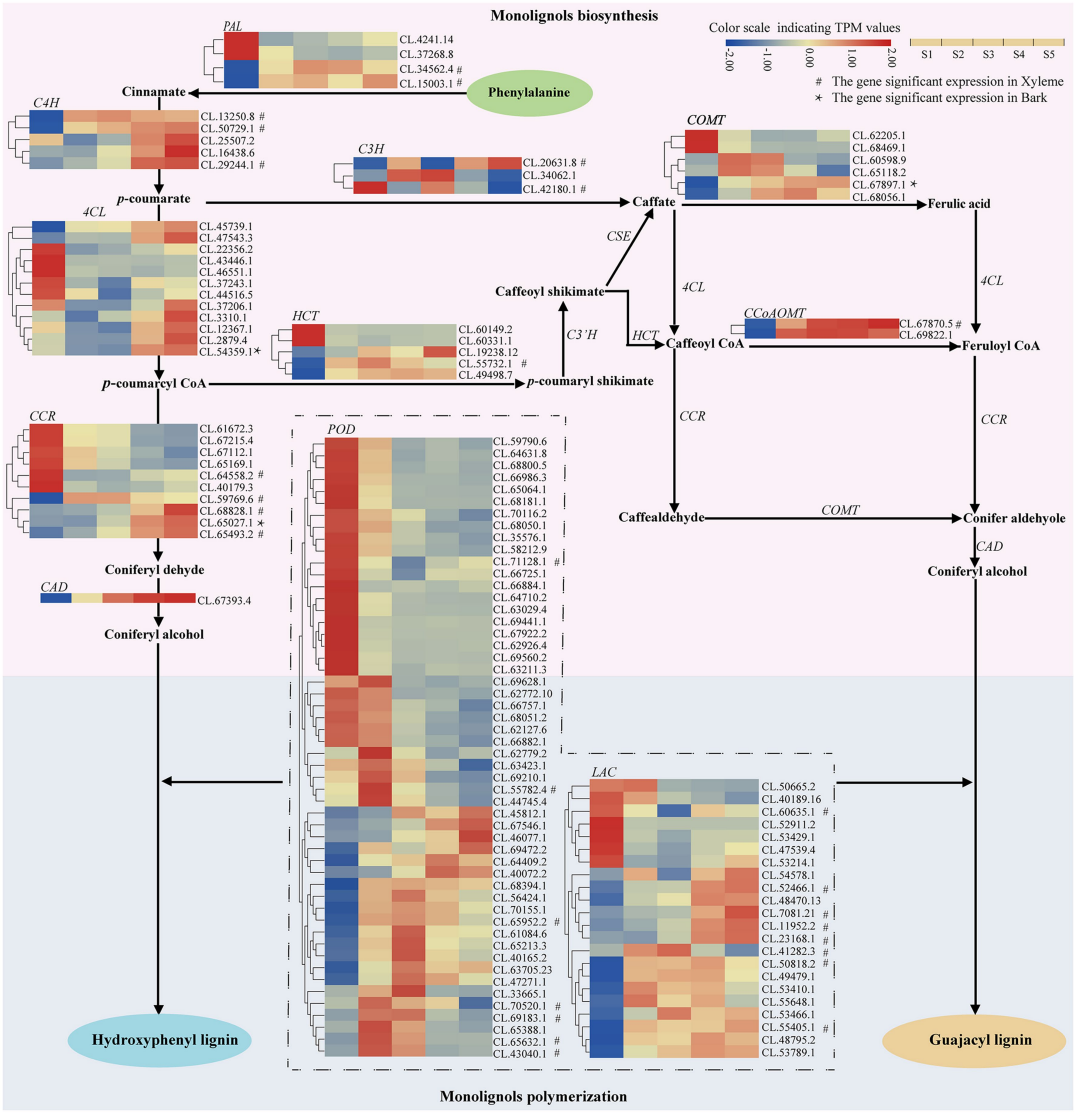
Heatmap depicting the expression profiles of DEGs involved in lignin biosynthesis in *C. lanceolata*.

With the increases of lignification from S1 to S5, 12 higher-expression (the average TPM > 100) enzyme-coding genes including one of *PAL*, *C4H*, *4CL*, and *HCT*; two of *CCR*, *POD*, *COMT*, and *SAMS* were found (Supplementary Table S4). Moreover, the expression patterns of 13 lignin biosynthesis enzyme-coding genes (three *LAC*; two of *C4H*, *CCR* and *POD*; one *4CL*, *CCoAOMT*, *CAD*, and *COMT*) appeared continuously upregulated from S1 to S5. Among these 13 genes, three *LAC*, two *C4H*, two *CCR*, one *CCoAOMT*, one *POD* also showed significant expressions in the xylem (Figure 3; Supplementary Table S4). Interestingly, as a higher-expression enzyme-coding gene, *C4H* (CL.50729.1) not only appeared continuously upregulated expression with the increases of lignification but also showed expressions significantly in the xylem. On the other hand, 16 continuously downregulated structural genes, including three *CCR*, one *LAC*, 11 *POD* and one *SAD*, also were identified in this study (Table 5). The above enzyme expression patterns that varied along with the stem developmental gradient were considered the most likely involved in the lignin biosynthesis of *C. lanceolata* (Supplementary Table S4).

### Construction of Gene Co-expression Network

**Figure 4:**
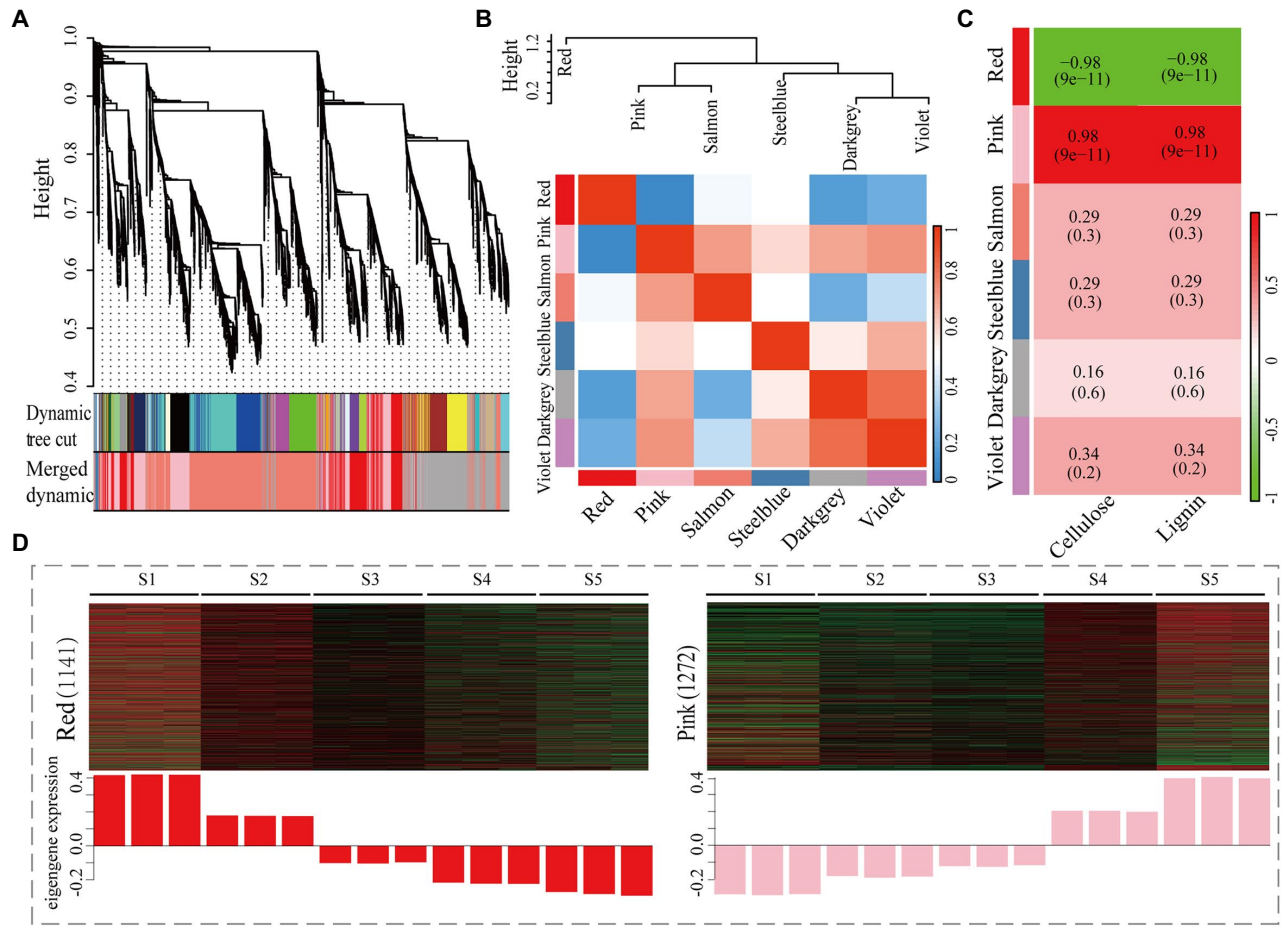
Co-expression analysis of 6,520 DEGs and cellulose/lignin contents in *C. lanceolata*. **(A)** Clustering dendrogram of DEGs, with dissimilarity based on the topological overlap, together with assigned module colors. **(B)** Analysis of connectivity of eigengenes in different modules with eigengenes (above) and the heatmap of connectivity of eigengenes (below). **(C)** Correlations of module and cellulose/lignin contents with corresponding *p*-values (in parentheses). **(D)** Expression profile of the genes in red **(A)** and pink **(B)** modules, respectively. Heatmaps showed the expression profiles of all the co-expressed genes (number given on the left) in the samples (labelled on top). The histogram shows the average eigengene expression among different samples.

To construct a regulatory network for those genes of cellulose/lignin biosynthesis pathway and identify specific genes that are highly correlated with cellulose/lignin biosynthesis in *C. lanceolata*, WGCNA was performed by using 6,520 DEGs (TPM > 1). As shown in Figure 4A, six different modules’ represented by branches with different colors were identified. Based on the modules connectivity effect, these six modules were classified into two groups, and according to that, the connectivity effect between modules is different (Figure 4B). A Pearson correlation coefficient analysis revealed that two co-expressed modules (red and pink) showed a strong correlation (|*r*| ≥ 0.8) with the biosynthesis of cellulose and lignin (Figure 4C). A heat map of each gene’s relative expression trend revealed that most of the genes in the pink module were highly expressed in S5 (highest lignified stem segment; Figure 4D). Conversely, most genes in the red module showed high expression levels in low lignified stem segments (S1 and S2; Figure 4D). Thereby, with the increase in lignification, DEGs with upregulated or downregulated expression trends were primarily assigned in pink and red modules, suggesting these genes might have participated in the biosynthesis of cellulose and lignin. Furthermore, functional enrichment analysis of DEGs in red and pink modules was performed. The genes in these two modules were significantly enriched to categories of catalytic activity (Supplementary Figure S6A) and molecular function (Supplementary Figure S6C) in the GO database, respectively. The top annotated KEGG terms were phenylpropanoid biosynthesis for the genes in red and pink modules (Supplementary Figures S6B,D). This pathway is one of the essential metabolic processes that determine the availability of lignin precursors (Wadenbäck et al., 2008). The annotation of the genes in red and pink modules suggests that the genes in these two modules may play important roles in the accumulation or biosynthesis of those two biopolymers.

### Transcription Regulatory Modules Involved in Cellulose and Lignin Biosynthesis

**Figure 5:**
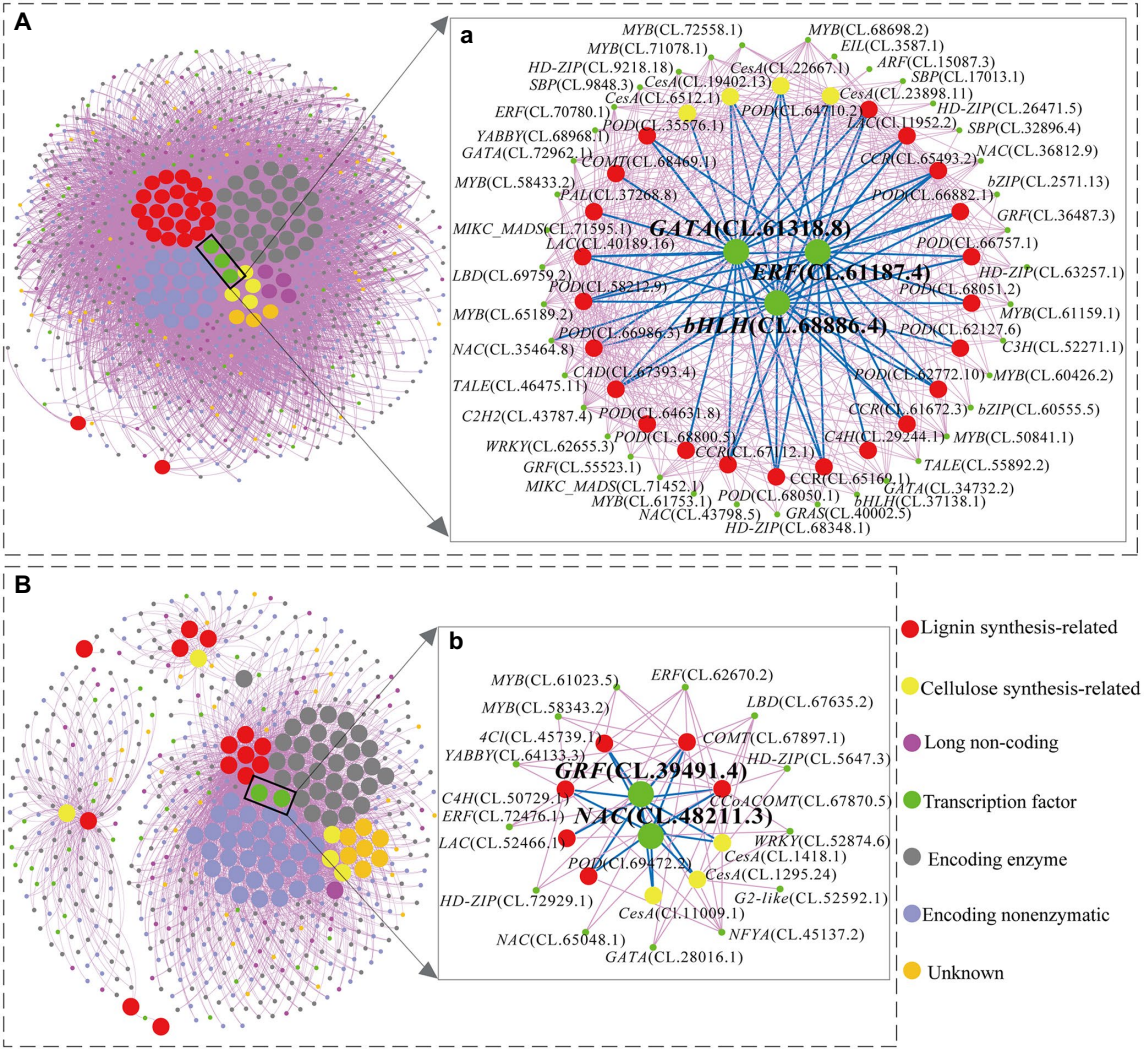
The genes regulatory network associated with cellulose and lignin biosynthesis in red **(A)** and pink **(B)** modules. (a): The directly and indirectly connected genes to the three hub TFs in red modules. (b): The directly and indirectly connected genes to the two hub TFs in pink modules.

The gene co-expression network analysis revealed a hierarchical organization of highly connected genes in the red and pink module, through which core controlling (hub) genes in the modular network could be identified. A total of 93 hub-genes were identified in the red module, in which only three transcription factors (TFs) were included [*GATA*, *ERF* (ethylene response factor), *bHLH* (basic helix–loop–helix)]. The expressions pattern of these three TFs continuously decreased with the increase in lignification (negative correlation; Supplementary Table S4) and co-expressed with 26 structural genes, which belong to the identified hub-genes in the red module (Figure 5A a). Interestingly, among the 26 structural genes, the expression pattern of *POD* (CL.35576.1, CL.58212.9, CL.66986.3, CL.62127.6, CL.66757.1, CL.66882.1 CL.64710.2, CL.67112.1), *LAC* (CL.40189.16), *CCR* (CL.65169.1, CL.61672.3) showed continuously downregulated in different stem developmental gradient (from S1 to S5).

On the other hand, a total of 91 hub-genes were identified in the pink module. However, only two hub-genes (CL.39491.4 and CL.48211.3) encoded TFs including one *NAC* (*NAM*, *ATAF1/2*, *CUC2*) and one *GRF* (*growth-regulating factor*) transcription factor. These two TFs are highly co-expressed with nine identified enzyme-coding genes in the cellulose and lignin biosynthesis pathway (Figure 5B b). In particular, with the enhancement of lignification (from S1 to S5), the expression patterns of *CesA* (CL.1295.24), *C4H* (CL.50729.1), *CCoAOMT* (CL.67870.5), *LAC* (CL.52466.1) were significantly upregulated and showed significant expression in the xylem (Supplementary Table S4; Figure 5B b). In addition, *C4H* (CL.50729.1) and *COMT* (CL.67897.1) also showed higher expression (the average TPM > 100) from S1 to S5 (Supplementary Table S4).

**Figure 6:**
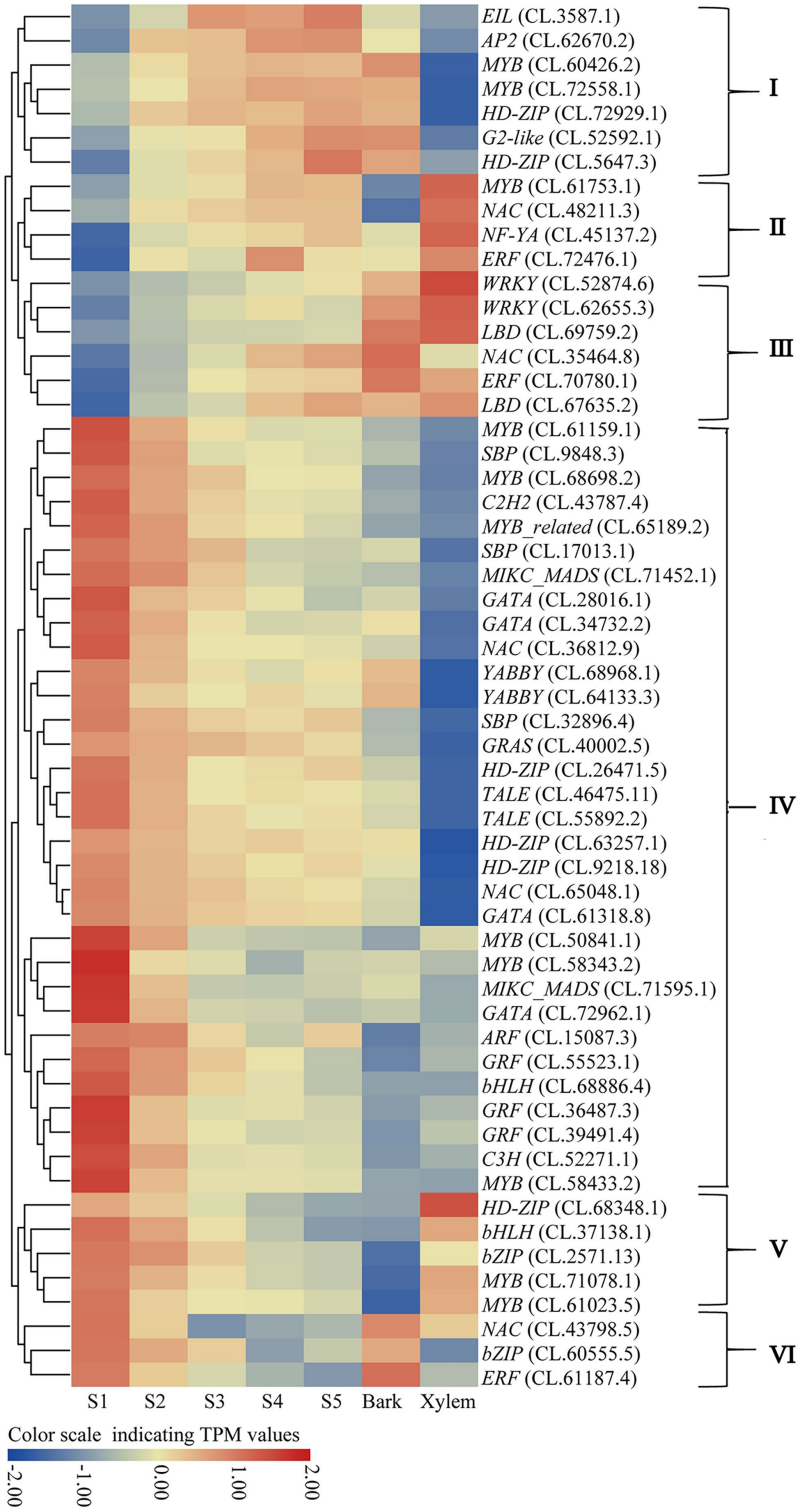
The expression patterns of identified TFs by WGCNA. (I) Upregulated expression from S1 to S5 and significant expression in bark; (II) upregulated from S1 to S5 and significant expression in xylem; (III) upregulated expression from S1 to S5 and no significant expression in bark and xylem; (IV) downregulated from S1 to S5 and no significant expression in bark and xylem; (V) downregulated from S1 to S5 and significant expression in xylem; (VI) downregulated from S1 to S5 and significant expression in bark.

It is worth noting that there were 39 and 13 TFs indirectly co-expressed with these hub TFs in the red and pink modules, respectively (Figure 5). These TFs’ expression patterns from S1 to S5, bark and xylem could be divided into six categories (I–VI; Figure 6). Among them, a total of 14.0% TFs showing upregulated from S1 to S5 have a significant expression in xylem (II and III) and 68.5% downregulated from S1 to S5 have a low expression in xylem (IV and VI). These TFs were probably involved in the regulatory network of lignin and cellulose biosynthesis. Combined with the preceding analysis in this study, eight TFs, including *ERF* (CL.61187.4), *GATA* (CL.61318.8), *NAC* (CL.43798.5; CL.48211.3), *LBD* (CL.69759.2), and *MYB* (CL.50841.1; CL.61023.5) were identified as the most likely candidate genes that involved in regulating the lignification process of *C. lanceolata*.

### The Expression Pattern of Regulatory Genes in Compression Wood

**Figure 7:**
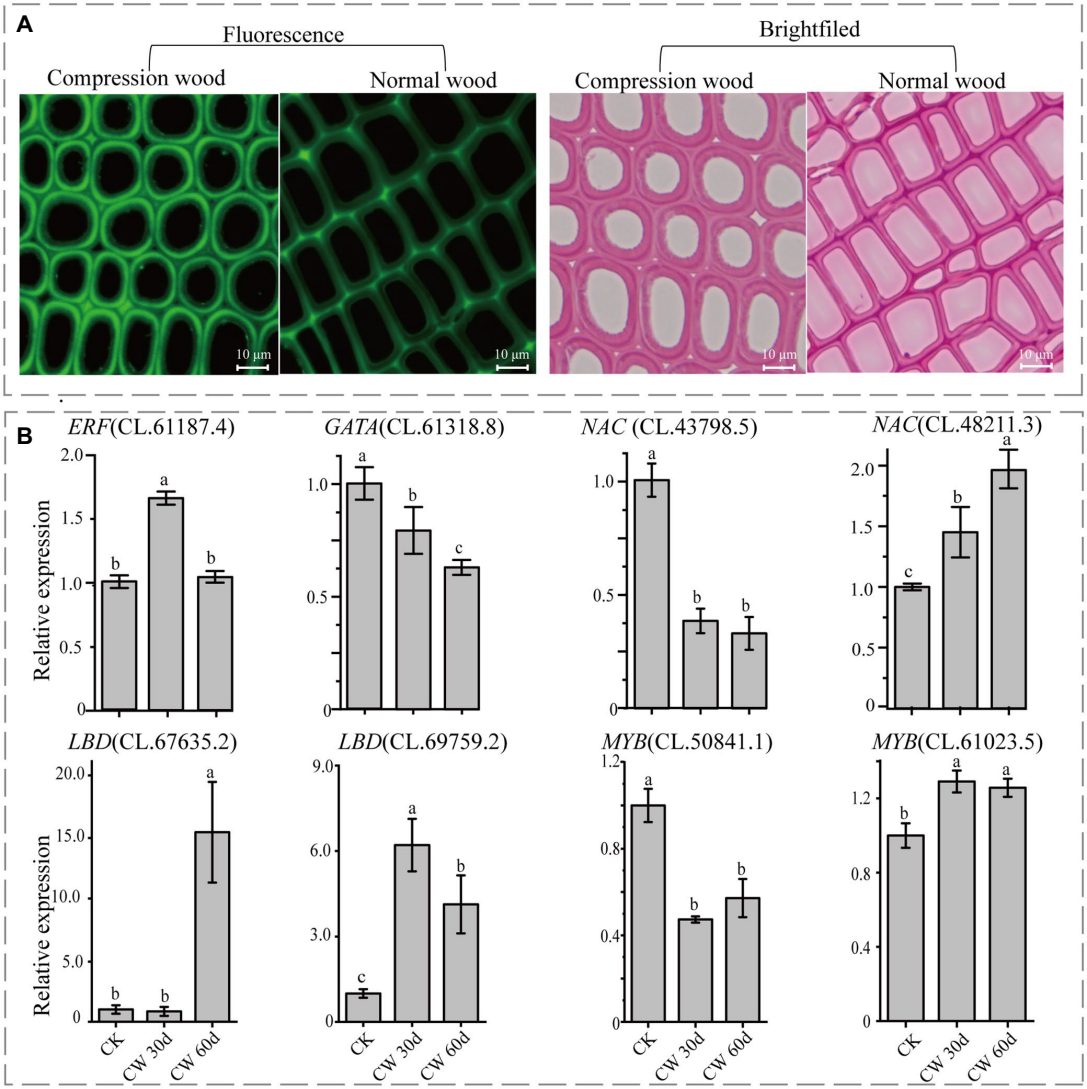
Changes of the cell wall structure and the relative transcriptional abundance of identified transcription factors. **(A)** Observation of compression wood and normal wood (30 days) by safranine staining and fluorescence microscopy. **(B)** The relative transcriptional abundance of eight TFs in normal wood (CK) and compression wood (CW). Values followed by different lower-case letters are significantly different according to Duncan test (*p* < 0.05).

Compression wood, a specialized reaction wood in response to gravity, could be used as an experimental model for wood formation. In this study, compression wood was induced to check the expression pattern of the identified TFs during wood formation. Compared with the normal wood, the tracheids became rounder in compression wood. Further, the secondary cell walls were thicker, and apparent intercellular spaces appeared between the corners of adjoining cells in the compression wood (Figure 7A). Moreover, autofluorescence of lignin detected by fluorescence microscopy showed that lignin content also increased in compression wood comparison to normal wood (Figure 7A). As these typical characteristics of compression wood were observed in *C. lanceolata*, it is rational to detect their involvement in wood formation for those identified genes.

**Figure 8:**
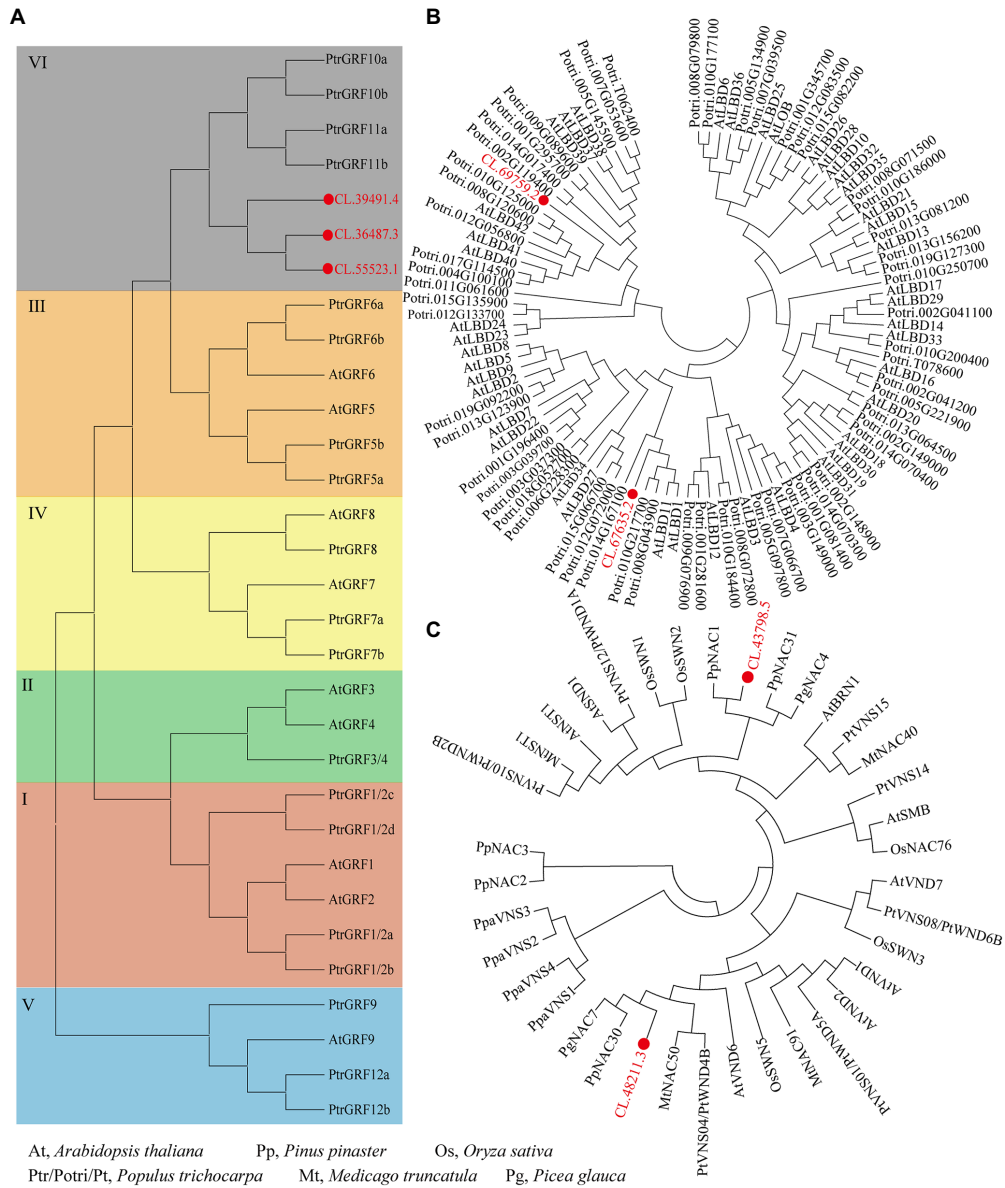
Phylogenetic analyses of *C. lanceolata* GRF, LBD and NAC TFs with their homologs from other plants. **(A)** Phylogenetic analysis of *C. lanceolata* GRF TFs with homologs from *Arabidopsis thaliana* and *Populus trichocarpa*; **(B)** phylogenetic analysis of *C. lanceolata* LBD TFs with homologs from *A. thaliana* and *P. trichocarpa*; **(C)** phylogenetic analysis of *C. lanceolata* NAC TFs with homologs from five plant species.

The qRT-PCR was performed to detect the expressions patterns of eight TFs (identified through WGCNA) in normal wood and 30 and 60 days of compression wood. Compared to normal wood, the expressions of *NAC* (CL.43798.5), *MYB* (CL.50841.1), and *GATA* (CL.61318.8) significantly decreased in compression wood, which probably means they were negative regulators (Figure 7B). The expressions of *NAC* (CL.48211.3), *MYB* (CL.61023.5), and *LBD* (CL.69759.2) increased significantly, which likely means that they were positive regulators during wood formation in *C. lanceolata* (Figure 7B). Interestingly, the expression of *LBD* (CL.67635.2) has not changed significantly in the 30 days compression wood but appeared substantial increases with the extension of induced time (60 days; Figure 7B). In addition, the expression of *ERF* (CL.61187.4) significantly increased in the early stage of induced compression wood (30 days) and then fell back to the same level as the normal wood (60 days; Figure 7B). The expression pattern analysis of these eight TFs in compression wood further illustrated that these unique TFs have a strong relationship with wood formation. In addition, among these five TFs families, three families were randomly selected for phylogenetic analysis to further demonstrate the relationship between identified TFs in *C. lanceolata* with the homologous genes proved to be involved in wood formation in other species. The phylogenetic analysis indicated that the identified *GRF*, *LBD* and *NAC* genes in *C. lanceolata* were clustered with homologs in the transcriptional regulation network of secondary cell wall formation in angiosperms (Figure 8).

Furthermore, 10 TFs, which were indirectly co-expressed with hub TFs, were randomly selected for expression analysis in the compression wood. As shown in Supplementary Figure S7, with the extension of induced time in compression wood experiment (means increased with the degree of lignification), these randomly selected genes indeed showed differential expression during compression wood formation (Supplementary Figure S7). These results further revealed that these regulatory genes identified by WGCNA were probably involved in regulation of wood formation.

## Discussion

### Generation of Comprehensive and High-Quality Transcriptomic Data of *Cunninghamia lanceolata*

High-quality genome and transcriptome sequences are indispensable for timber tree molecular breeding. In this study, the transcriptome of *C. lanceolata* was sequenced by SMRT for the first time, which generated 616,778 full-length non-chimeric sequences with an average size of 2,450 bp according to 20 Gb sequencing data. This research goes far beyond our previous work, in which only 83,248 assembled sequences with an average length of 449 bp were obtained based on 3.62 G SGS data (Huang et al., 2012). Similarly, in other gymnosperms like *Ginkgo biloba*, Ye et al. (2019) have developed 23.36 Gb transcriptome data and identified 120,616 consensus isoform sequences (with a mean length of 2,487 bp). Likewise, Mei et al. (2020) reported that 481,602 circular consensus sequences (with an average length of 2051 bp) were generated from high-yield clones of *P. massoniana*. Higher sequencing depth and long sequencing reads of our study provided longer and more accurate unassembled transcripts, which resulted in a comprehensive and high-quality transcriptome of *C. lanceolata*. Up to date, this study provided the most abundant genetic information resources for *C. lanceolata*, significantly contributing to further study of this valuable timer species.

### Enzyme-Coding Genes Involved in Cellulose and Lignin Biosynthesis

With the stem development gradient of *C. lanceolata* (from S1 to S5), the content of cellulose and lignin has increased significantly (Table 1), and we identified that 28 and 125 enzyme-coding genes are involved in cellulose and lignin biosynthesis, respectively. Sequence analysis indicated that these genes were homologous to genes with functions in secondary cell wall synthesis in previous studies. For example, in compressive wood experiments of *Betula luminifera*, the *BlKOR1*, a homologous gene of *AtKOR1*, was significantly upregulated during the early stages of tension wood (TW) formation (Cai et al., 2018). In this study, *ClKOR* (CL.32747.44) was also a homologous gene of *AtKOR1*, showing the same expression trend at the early stages of TW formation, which probably means that their functions are similar in trees. Meanwhile, five *CesA* genes of *C. lanceolata* were found to be significantly upregulated along with the development gradient of the stem and its homologous genes in *Populus tremula* × *Populus tremuloides* (Andersson-Gunnerås et al., 2006), *Eucalyptus globulus* (Djerbi et al., 2005) and *B. luminifera* (Cai et al., 2018) also had the same expression pattern. In addition, the identified *FRK* gene in *C. lanceolata* also had a similar tissue-specific expression pattern to its homologous gene in other plants (Cao et al., 2018). Therefore, our work further revealed the potential roles of these enzyme-coding genes in cellulose biosynthesis and provided more candidate genes for the genetic improvement of wood properties in *C. lanceolata*.

Lignin is a kind of complex and variable structure polymerized from monolignols (Vanholme et al., 2019), and the lignin in conifers is mainly composed of guaiacyl (G) and hydroxyphenyl (H) monolignols (Lange et al., 1995; Gindl, 2002). However, in angiosperms, the lignin is mainly composed of syringyl (S) monolignols, and *SAD* was regarded as a key enzyme for S monolignol biosynthesis (Li et al., 2001). In this study, only one *SAD* (CL.67393.4) was identified. Its expression pattern appeared continuously decreasing with the increases in lignification, which probably implied that this *SAD* gene is responsible for the limited S monolignol biosynthesis in the xylem of *C. lanceolata*. On the other hand, most enzyme-coding genes (e.g., *C4H*, *CAD* and *CCoAOMT*), which were directly associated with the synthesis of G- and H-monolignol, have upregulated expressions with the increases in lignification. Therefore, the identified genes related to specific biological processes of lignin were the potential targets for molecular breeding of *C. lanceolata*, which possibly help alter the content and composition of lignin to extend its industrial application (e.g., facilitate delignification in kraft pulping).

### The Expression Pattern of Regulatory Genes in Cellulose and Lignin Biosynthesis of *Cunninghamia lanceolata*

Many TFs already have been proven to be involved in controlling plants secondary wall formation in herbaceous (e.g., *Arabidopsis*) and woody plants (e.g., *Populus*; Kim et al., 2020; Li et al., 2022). In *Arabidopsis*, a multi-level regulatory network has been established in which the TFs of *NAC* and *MYB* act as core members to participate in controlling the formation of the secondary wall (Zhong et al., 2007; Taylor-Teeples et al., 2015). During the formation of the secondary cell wall (SCW), generally, there are three kinds of regulators: (1) *NAC* TFs [e.g., *VND* (*vascular-related NAC-domain*)], which were considered as secondary wall *NAC* (SWNs) master switches; (2) *MYB46/83*; (3) *MYB* family members, e.g., *MYB58/63/85* (Zhong et al., 2007, 2008; Zhou et al., 2009). In addition, other TFs, *LBD*, and *bHLH*, have also been proved to be involved in the regulatory network of SCW. In this study, the homologous TFs at different levels of the regulatory network in angiosperms have been found in *C. lanceolata*, suggesting that there is probably a comparable secondary wall development regulation network in gymnosperms.

*NAC* TFs are considered one of the essential regulators of wood formation and have already been discovered as the switches of secondary wall formation (Akiyoshi et al., 2021; Kim et al., 2021). In this study, a series of *NAC* TFs that are homologous to the SWN of *Arabidopsis* were found to be co-expressed with the enzyme-coding genes related to cellulose/lignin biosynthesis. Among them, CL.482111.3 was highly expressed in the xylem (Figure 7). The compressed wood experiment further showed that CL.48211.3 was significantly expressed with the increases in lignification (30 and 60 days). Based on phylogenetic analysis, CL.48211.3 was clustered with *AtVND6* from *Arabidopsis* and *PtWND4B* (wood-associated *NAC* domain) from *Populus tomentosa* (Figure 8C). In *Arabidopsis*, *AtVND6* was upregulated during the formation of vessel molecules in isolated xylem, and overexpression caused thickening of epigenetic xylem (Kubo et al., 2005). Besides, *PtWND4B* of *P. tomentosa* was also highly expressed in lignified stem segments (Zhong et al., 2010). Therefore, as a homolog of *AtVND6* and *PtWND4B*, CL.48211.3 is also probably a positive regulator in the lignification of *C. lanceolata*.

In addition, CL.43798.5 was clustered with *PpNAC1* from *Pinus pinaster* (Figure 8C). *PpNAC1* was found to be upregulated and had higher expression in the secondary xylem, which was considered as a positive regulatory transcription factor in the lignification process (Pascual et al., 2017). However, contrary to the above results, CL.43798.5 has downregulated expression in compressed wood. One possible explanation is that isomers of CL.43798.5 may exist in *C. lanceolata* and play an antagonistic role in the process of lignification. For example, alternative splicing events in *NAC* TFs are widespread and have become an essential regulatory mechanism in biological development (Irimia et al., 2007). *PtWND1B* had an alternative splicing event in secondary xylem fiber cells, and two isoforms demonstrated antagonistic effects for cell wall thickening (Zhao et al., 2014).

As one of the largest transcription factor families in the plants, *MYB* TFs play key roles in regulating the phenylpropanoid pathway and the biosynthesis of cellulose/lignin (Golfier et al., 2021; Xiao et al., 2021). In this study, four *MYB* TFs (CL.50841.1, CL.61023.5, CL.61753.1, and CL.71078.1) were preferentially expressed in the xylem. Notably, *MYB4* (CL.71078.1) and *MYB49* (CL.61753.1) had already been proved that they might be involved in the process of lignification (Zhuang et al., 2021). *LBD* is also a specific TFs family in the plants, and several members of this family play essential roles in the development of plant secondary wall. In this study, two *LBD* transcription factors CL.67635.2 and CL.69759.2 were identified. CL.67635.2 was upregulated and significantly has higher expressed in secondary xylem. Phylogenetic analysis further showed that CL.67635.2 was clustered with *PagLBD3* (Potri.008G043900) from *Poplar* (Figure 8B). *PagLBD3* is highly expressed in wood-forming tissues, and overexpression of *PagLBD3* promoted the secondary growth of poplar stem (Han et al., 2021). This implies that homologs of *PagLBD3*, like CL.67635.2 might also play an important role in wood formation. In addition, *LBD* transcription factor CL.69759.2 had the same expression pattern in this study.

In addition, we found other TFs like *GRF*, *GATA*, *bHLH*, and *ERF*, also probably involved in the development of the secondary wall of *C. lanceolata*. For example, three *GRF* TFs were preferentially expressed in xylem, and the phylogenetic analysis further showed that they were clustered with *PtrGRF10a*, *PtrGRF10b*, *PtrGRF11a*, and *PtrGRF11b* (Figure 8A). Additionally, the homologous gene of the *GATA* transcription factor found in this study has already been proved to be involved in the wood formation of *P. tomentosa*. Moreover, *PtrGATA12* was preferentially expressed in developing secondary xylem, and the contents of hemicellulose and lignin, as well as the thickness of fibers in overexpressed transgenic plants, were also changed (Ren et al., 2021). In this study, a series of regulatory factors (i.e., *NAC*) and downstream-regulated factors (i.e., *MYB*) involved in the secondary development of stems were identified. These results not only help us to understand the regulation process of lignin/cellulose biosynthesis in gymnosperm but also would be conducive to developing purposeful genetic breeding.

## Conclusion

In this study, a comprehensive and valuable full-length transcriptome database of *C. lanceolata* was generated for the first time. Among the 48,846 high-quality full-length transcripts generated, 18,714 differentially expressed genes (DEGs) were identified along the stem developmental gradient. Furthermore, a total of 153 candidate enzyme-coding and 57 regulatory genes involved in cellulose and lignin biosynthesis were identified by using structural gene analysis and WGCNA. In addition, the identified transcription factors were further investigated during compression wood formation experimental to verify their relationships with wood formation. In conclusion, our study not only presents a useful genetic resource of *C. lanceolata* to study secondary wall biosynthesis, also will be beneficial for further molecular-assisted selection in *C. lanceolata* breeding.

## Data Availability Statement

The datasets presented in this study can be found in online repositories. The names of the repository/repositories and accession number(s) can be found at: the raw sequence data (SMRT and SGS) reported in this paper have been deposited in the Genome Sequence Archive (Genomics, Proteomics and Bioinformatics 2021) in National Genomics Data Center (Nucleic Acids Res 2021), China National Center for Bioinformation/Beijing Institute of Genomics, Chinese Academy of Sciences, SMRT sequencing data under accession number CRA003755 that are publicly accessible at: https://ngdc.cncb.ac.cn/gsa. SGS sequencing data under accession numbers CRA005211 that are publicly accessible at: https://ngdc.cncb.ac.cn/gsa.

## Author Contributions

HH and EL performed conceptualization, supervision, and funding acquisition. X-GH and HZ did methodology and visualization. HZ provided software. X-GH, HZ, and MD done validation. SG performed formal analysis. PB investigated the study. X-GH, HZ, MD, SG, and PB were involved in data curation. X-GH done writing-original draft preparation. X-GH, EL, TW, and ZT contributed to writing—review and editing. HH done project administration. All authors contributed to the article and approved the submitted version.

## Funding

This work was supported by the Key Research and Development Project of Zhejiang Province (2021C02054) and Zhejiang Science and Technology Major Program on Agricultural New Variety Breeding (2021C02070-8).

## Conflict of Interest

The authors declare that the research was conducted in the absence of any commercial or financial relationships that could be construed as a potential conflict of interest.

## Publisher’s Note

All claims expressed in this article are solely those of the authors and do not necessarily represent those of their affiliated organizations, or those of the publisher, the editors and the reviewers. Any product that may be evaluated in this article, or claim that may be made by its manufacturer, is not guaranteed or endorsed by the publisher.

## Supplementary Material

The Supplementary Material for this article can be found online at: https://www.frontiersin.org/articles/10.3389/fpls.2022.883720/full#supplementary-material
