# Supplementary figures and images for: Full-Length Transcriptome Sequencing and Comparative Transcriptomic Analyses Provide Comprehensive Insight Into Molecular Mechanisms of Cellulose and Lignin Biosynthesis in Cunninghamia lanceolata
Source: Front Plant Sci. 2022 May 31;13:883720. doi: 10.3389/fpls.2022.883720 (PMC9194830; doi:10.3389/fpls.2022.883720)

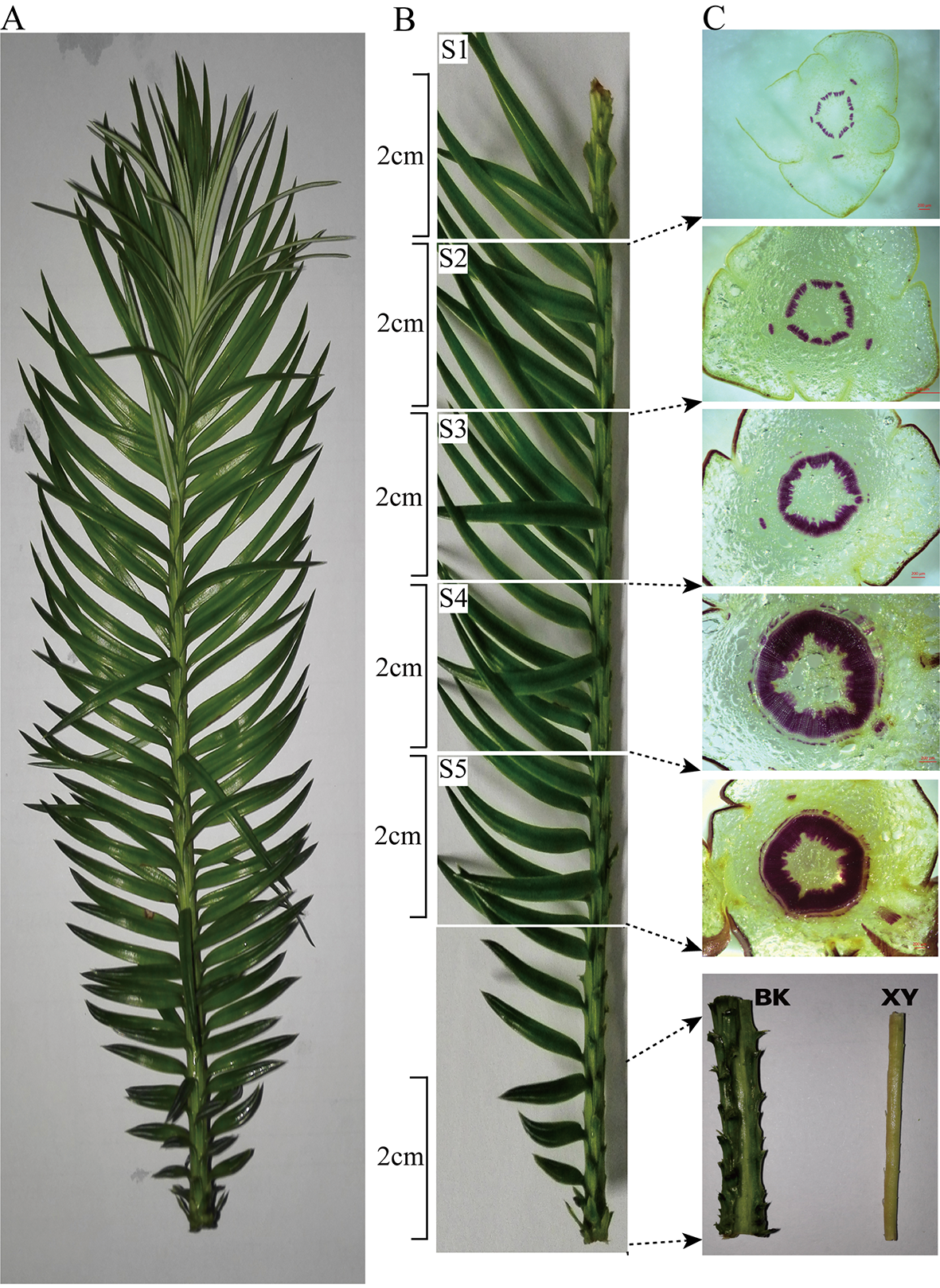

Supplement: Supplementary file 5 [file Image_1.TIF]

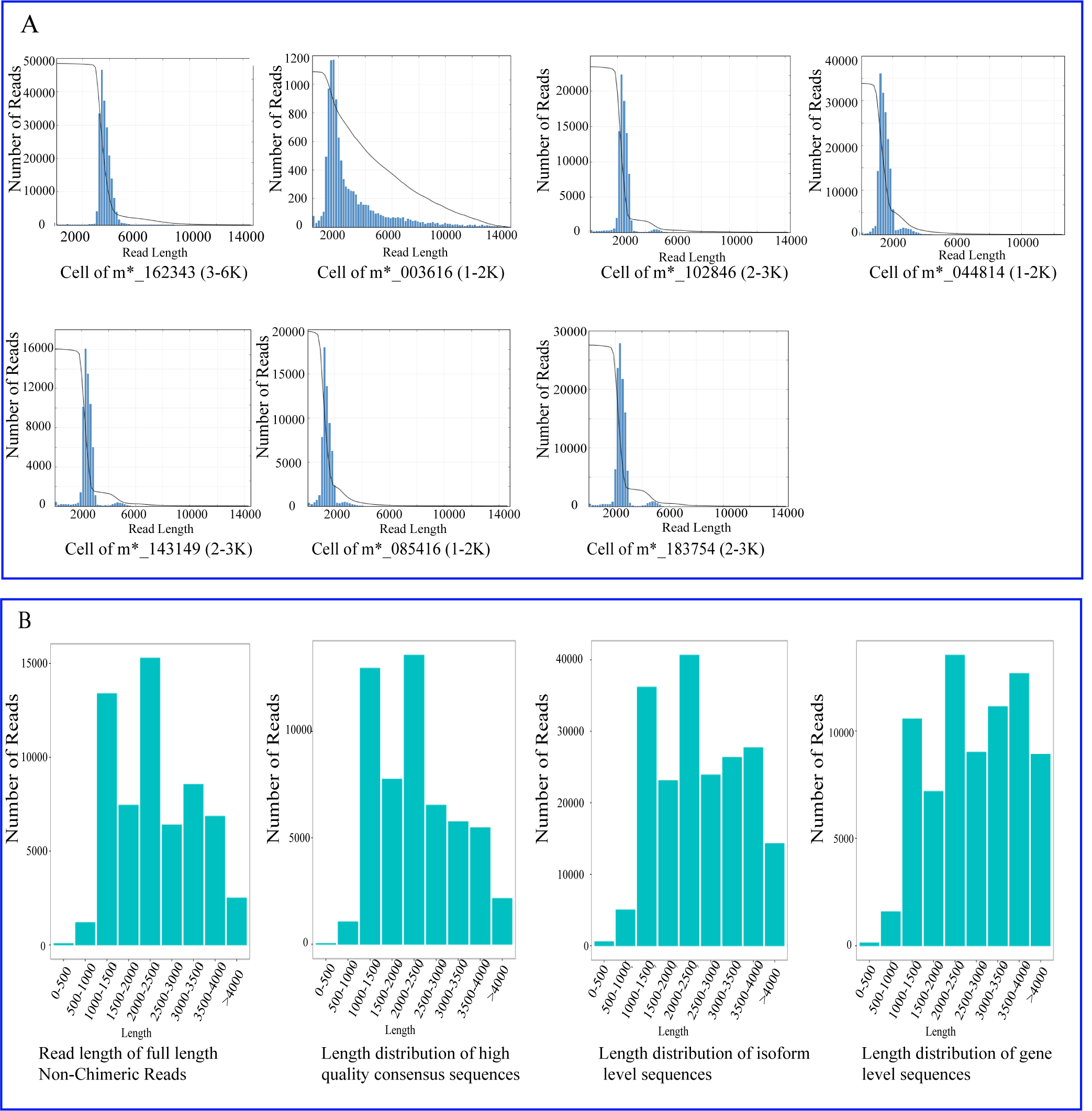

Supplement: Supplementary file 6 [file Image_2.TIF]

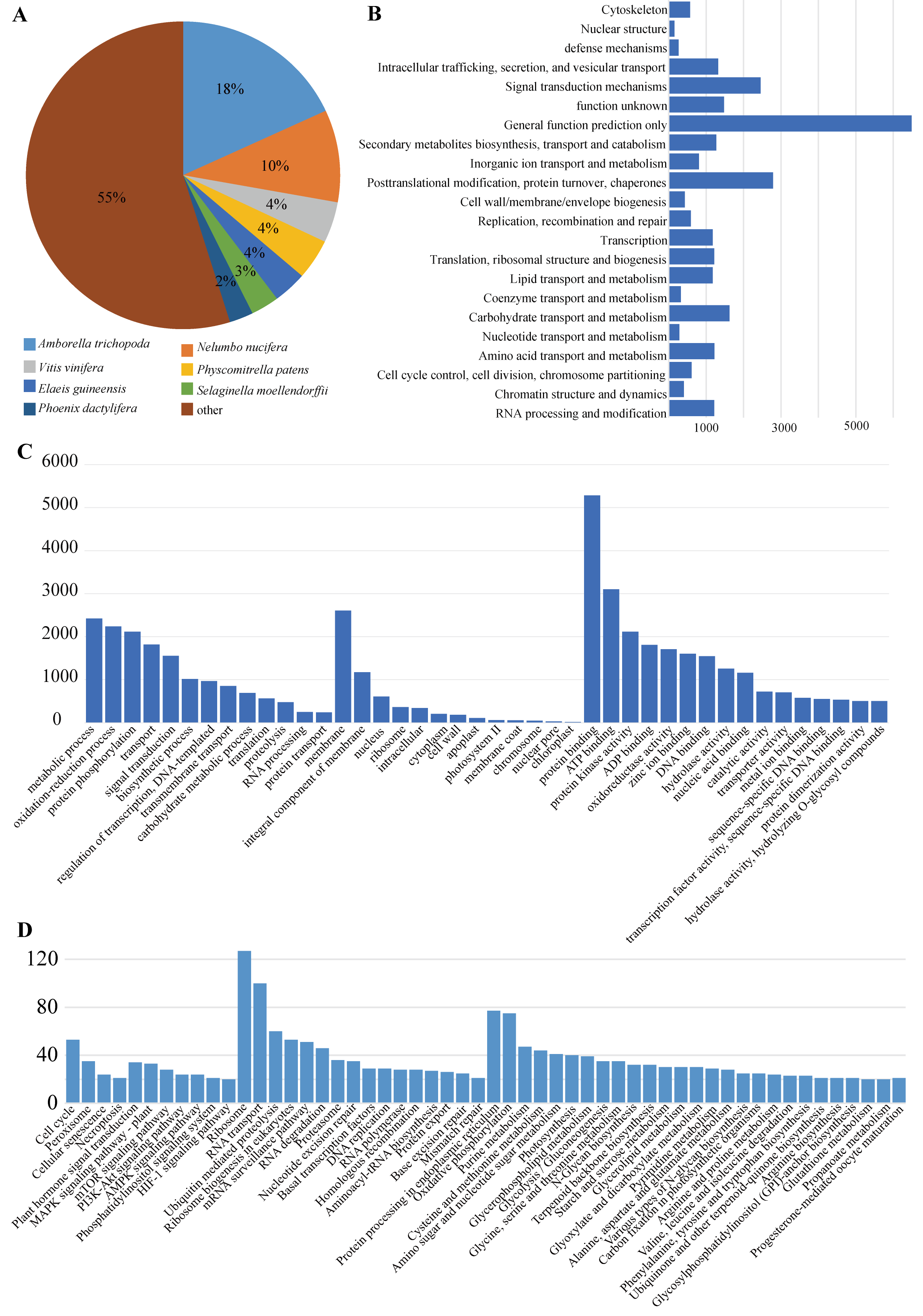

Supplement: Supplementary file 7 [file Image_3.TIF]

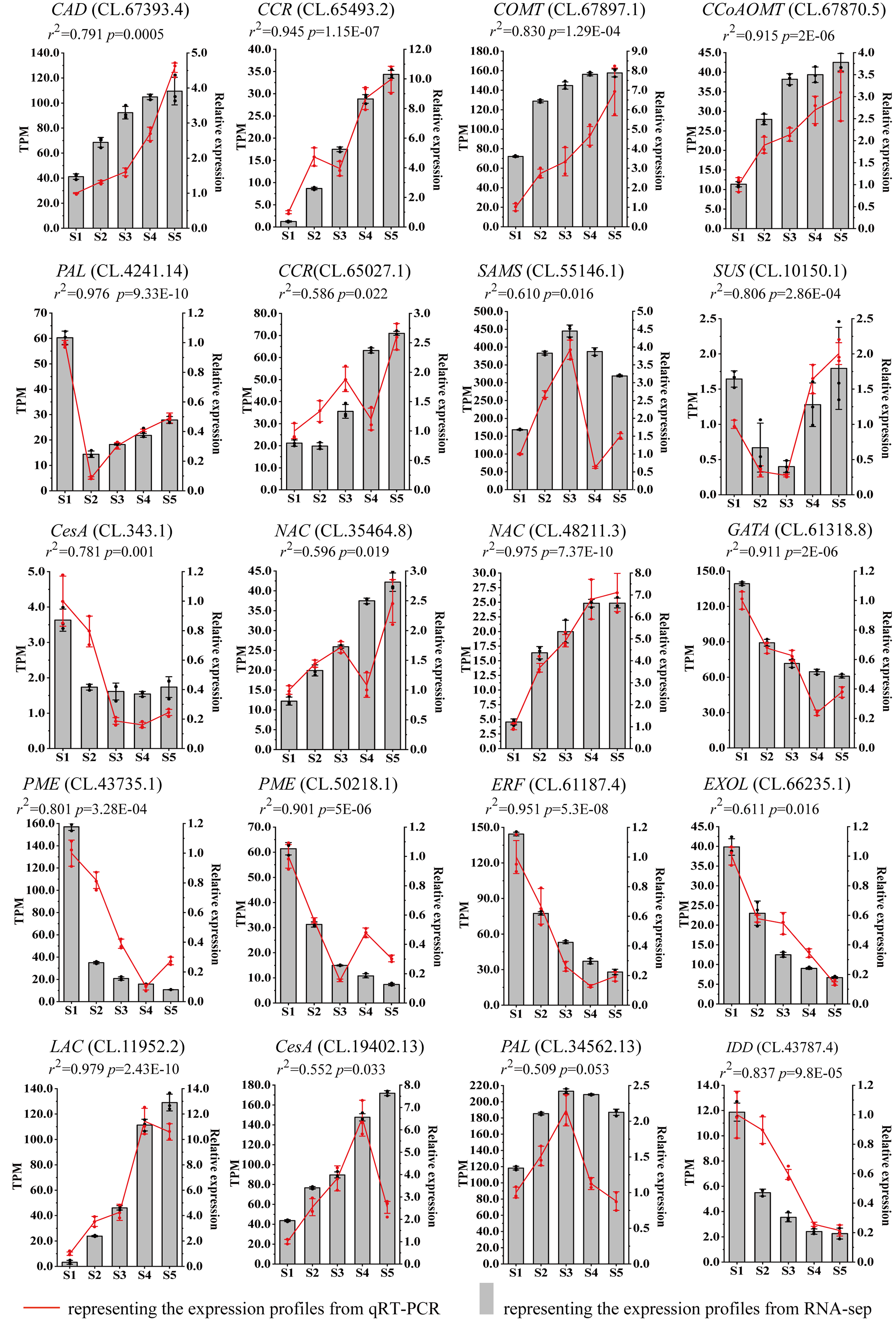

Supplement: Supplementary file 8 [file Image_4.TIF]

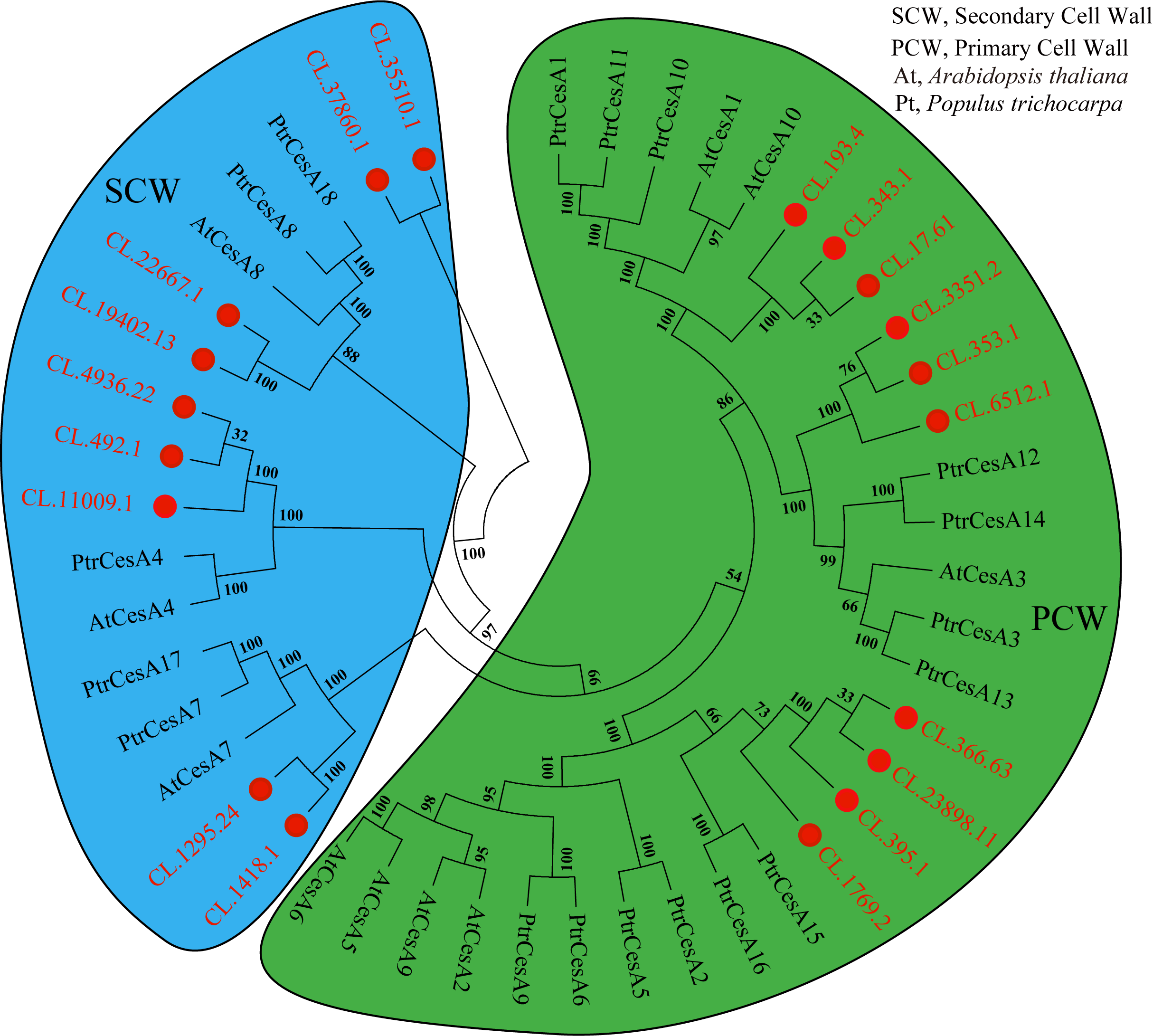

Supplement: Supplementary file 9 [file Image_5.TIF]

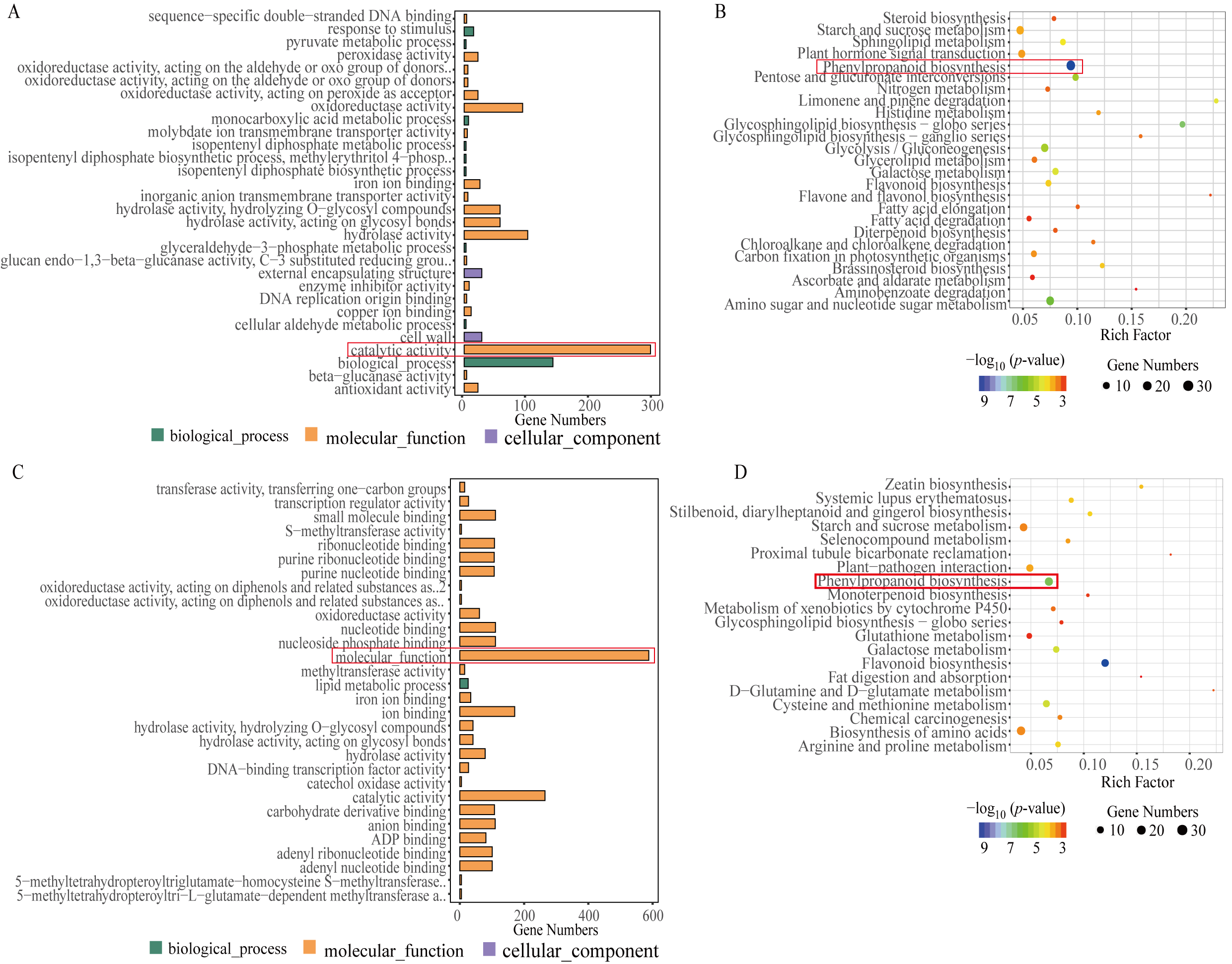

Supplement: Supplementary file 10 [file Image_6.TIF]

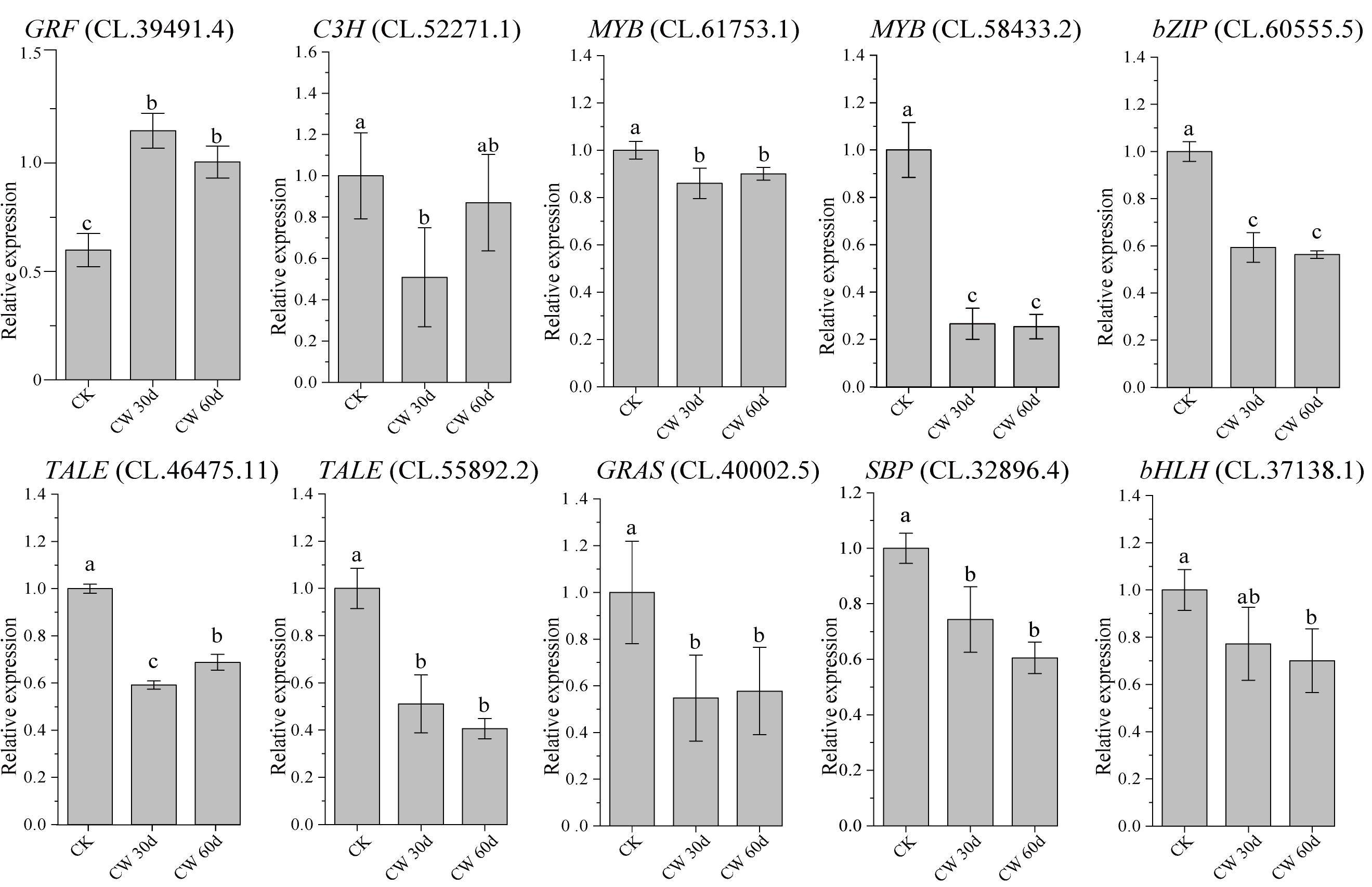

Supplement: Supplementary file 11 [file Image_7.TIF]
